# Nicotinamide riboside improves muscle mitochondrial biogenesis, satellite cell differentiation, and gut microbiota in a twin study

**DOI:** 10.1126/sciadv.add5163

**Published:** 2023-01-13

**Authors:** Helena A. K. Lapatto, Minna Kuusela, Aino Heikkinen, Maheswary Muniandy, Birgitta W. van der Kolk, Swetha Gopalakrishnan, Noora Pöllänen, Martin Sandvik, Mark S. Schmidt, Sini Heinonen, Sina Saari, Juho Kuula, Antti Hakkarainen, Janne Tampio, Tuure Saarinen, Marja-Riitta Taskinen, Nina Lundbom, Per-Henrik Groop, Marja Tiirola, Pekka Katajisto, Marko Lehtonen, Charles Brenner, Jaakko Kaprio, Satu Pekkala, Miina Ollikainen, Kirsi H. Pietiläinen, Eija Pirinen

**Affiliations:** ^1^ Obesity Research Unit, Research Program for Clinical and Molecular Metabolism, Faculty of Medicine, University of Helsinki, FIN-00014 Helsinki, Finland.; ^2^ Research Program for Clinical and Molecular Metabolism, Faculty of Medicine, University of Helsinki, FIN-00014 Helsinki, Finland.; ^3^ Institute for Molecular Medicine Finland (FIMM), HiLIFE, University of Helsinki, Helsinki, Finland.; ^4^ Institute of Biotechnology, HiLIFE, University of Helsinki, Helsinki, Finland.; ^5^ Department of Biochemistry, Carver College of Medicine, University of Iowa, Iowa City, IA 52242, USA.; ^6^ Department of Radiology, Medical Imaging Center, Helsinki University Hospital and University of Helsinki, Helsinki, Finland.; ^7^ Population Health Unit, Finnish Institute for Health and Welfare, Helsinki, Finland.; ^8^ Population Health Unit, Finnish Institute for Health and Welfare, Oulu, Finland.; ^9^ School of Pharmacy, University of Eastern Finland, Kuopio, Finland.; ^10^ Abdominal Center, Department of Gastrointestinal Surgery, Helsinki University Hospital, Helsinki, Finland.; ^11^ Folkhälsan Institute of Genetics, Folkhälsan Research Center, Helsinki, Finland.; ^12^ Abdominal Center, Nephrology, Helsinki University Hospital and University of Helsinki, Helsinki, Finland.; ^13^ Department of Diabetes, Central Clinical School, Monash University, Melbourne, Australia.; ^14^ Department of Environmental and Biological Sciences, University of Jyväskylä, FI-40014 Jyväskylä, Finland.; ^15^ Department of Cell and Molecular Biology, Karolinska Institutet, Stockholm, Sweden.; ^16^ Department of Diabetes and Cancer Metabolism, City of Hope National Medical Center, Duarte, CA 91010, USA.; ^17^ Faculty of Sport and Health Sciences, University of Jyväskylä, FI-40014 Jyväskylä, Finland.; ^18^ Minerva Foundation Institute for Medical Research, Helsinki, Finland.; ^19^ Abdominal Center, Healthy Weight Hub, Helsinki University Hospital and University of Helsinki, Helsinki, Finland.; ^20^ Research Unit of Biomedicine and Internal Medicine, Faculty of Medicine, University of Oulu, FIN-90220 Oulu, Finland.; ^21^ Medical Research Center Oulu, Oulu University Hospital and University of Oulu, Oulu, Finland.

## Abstract

Nicotinamide adenine dinucleotide (NAD^+^) precursor nicotinamide riboside (NR) has emerged as a promising compound to improve obesity-associated mitochondrial dysfunction and metabolic syndrome in mice. However, most short-term clinical trials conducted so far have not reported positive outcomes. Therefore, we aimed to determine whether long-term NR supplementation boosts mitochondrial biogenesis and metabolic health in humans. Twenty body mass index (BMI)–discordant monozygotic twin pairs were supplemented with an escalating dose of NR (250 to 1000 mg/day) for 5 months. NR improved systemic NAD^+^ metabolism, muscle mitochondrial number, myoblast differentiation, and gut microbiota composition in both cotwins. NR also showed a capacity to modulate epigenetic control of gene expression in muscle and adipose tissue in both cotwins. However, NR did not ameliorate adiposity or metabolic health. Overall, our results suggest that NR acts as a potent modifier of NAD^+^ metabolism, muscle mitochondrial biogenesis and stem cell function, gut microbiota, and DNA methylation in humans irrespective of BMI.

## Teaser

Nicotinamide riboside is a potent modifier of mitochondrial metabolism, satellite cell differentiation and gut microbiota.

## INTRODUCTION

Activation of mitochondria is an attractive treatment option for obesity and its related metabolic complications (*1*). Increasing intracellular levels of nicotinamide adenine dinucleotide (NAD^+^), the crucial cofactor for mitochondrial energy production, has shown great promise to improve mitochondrial number and oxidative capacity and to combat obesity and its related diseases in mice (*2*). This observation has generated a high interest on whether mitochondrial dysfunction and metabolic health can be improved by NAD^+^ boosters in humans.

The intracellular NAD^+^ levels can be increased by supplementation with NAD^+^ precursors (*2*). Vitamin B3 forms niacin (nicotinic acid), nicotinamide (NAM), and nicotinamide riboside (NR) are naturally occurring precursors of NAD^+^. They are converted to NAD^+^ via their distinct biosynthesis routes, Preiss-Handler and salvage pathways. The clinical use of niacin and NAM is challenged by dose-dependent adverse effects such as hepatotoxicity (*3*). Niacin also induces cutaneous flushing through activation of G protein–coupled receptor 109A (*4*). However, our previous study established that niacin can improve systemic NAD^+^ levels and muscle mitochondrial metabolism in humans without severe side effects (*5*). The newest vitamin B3 family member NR (*6*) has been shown to increase mitochondrial biogenesis and function, protect against diet-induced obesity and insulin resistance, and improve the gut microbiota composition in mice (*7*–*13*). Therefore, NR has rapidly entered clinical trials.

To date, clinical studies have shown NR to be safe and to raise NAD^+^ levels in whole blood, peripheral blood mononuclear cells, urine, muscle, and brain with a dose from 300 mg/day up to 2000 mg/day (*14*–*20*). Unfortunately, most human trials have failed to show substantial improvements in adiposity and insulin sensitivity in healthy overweight or obese individuals after a 3- to 12-week NR supplementation (*14*–*19*). However, improved physical performance, muscle acetylcarnitine levels, arterial stiffness, and blood pressure or decreased circulating inflammatory cytokines have been reported in a few studies with a short-term follow-up (*18*, *19*, *21*). At present, the evidence of the efficacy of NR on tissue mitochondrial biogenesis and function still relies on data on mice as positive outcomes are lacking from clinical studies (*19*, *22*). Until now, the number of human intervention studies with NR is still relatively low and the longest clinical trial conducted has been 12 weeks. Given that the effects of NR could be tissue specific and depend on body mass index (BMI), genetic background, and treatment duration, deep metabolic phenotyping of multiple tissues in well-controlled human interventions of longer duration is required.

We hypothesized that long-term NR supplementation could improve mitochondrial biogenesis in metabolically active tissues in humans. Therefore, we examined the effect of long-term supplementation of NR (5 months) not only on muscle and white adipose tissue (WAT) mitochondrial biogenesis (primary outcome) but also on body composition and multiple measures of metabolic health. Our design was unique: rare monozygotic (MZ) twin pairs who were discordant for BMI, allowing us to investigate whether the NR response differs in the leaner and the heavier cotwins with matched genetic background. In addition, we applied the cotwin control strategy in a small cohort of BMI-concordant MZ pairs to compare the effects of NR (one cotwin) to placebo (the other cotwin). We found that 5-month NR supplementation improves muscle mitochondrial biogenesis, muscle myoblast differentiation, and the gut microbiota composition and modulates DNA methylation, with potential effects on epigenetic control of gene expression, in both cotwins from the BMI-discordant pairs, i.e., regardless of BMI. However, these changes were not translated into improvements in adiposity or metabolic status. Together, our study underscores that long-term NR supplementation can affect various metabolic processes in humans.

## RESULTS

### Characteristics of study participants 

**Fig. 1.**
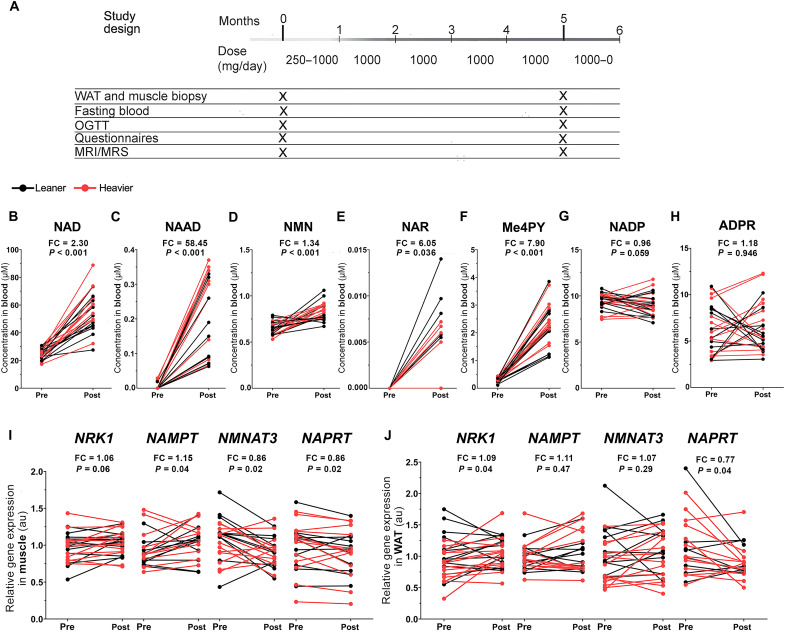
NR increases whole-blood NAD^+^ levels and tissue NAD^+^ biosynthesis in the twins from the BMI-discordant pairs. (**A**) Study protocol. The daily NR dose was gradually escalated from 250 mg/day by 250 mg/week to achieve the final treatment dose of 1 g/day. At the end of the study, the dose was decreased by 250 mg/week. Clinical examinations and collection of fasting blood samples and muscle and WAT biopsies were performed at baseline and after 5 months. (**B** to **H**) Whole-blood NAD metabolite levels before versus after NR (*n* = 14 twin pairs/28 individuals). (**I** and **J**) Expression of genes involved in NAD^+^ biosynthesis in muscle (I) and WAT (J) before versus after NR (*n* = 10 to 13 twin pairs/21 to 26 individuals). Arbitrary unit (au) indicates the relative target gene expression normalized to the expression of reference genes. Lines connect the pre- and post-values of each individual, with black denoting the leaner and red denoting the heavier cotwins. Fold change (FC) indicates the mean of the post-NR value divided by the pre-NR value. *P* values were calculated using paired Wilcoxon signed-rank test. See also Table 2 and figs. S1 and S2.

Together, 20 BMI-discordant (within-pair difference in BMI, <2.5 kg/m^2^) and 4 BMI-concordant MZ twin pairs, aged ~40 (interquartile range, 33 to 41), participated in the study (Table 1). Four BMI-discordant twin pairs discontinued the intervention (fig. S1). Women accounted for 44 and 50% of the analyzed BMI-discordant and BMI-concordant MZ twin pairs, respectively (Table 1). All twins from the BMI-discordant pairs were supplemented with NR. Of the concordant twin pairs (Table 1), one cotwin was randomized to placebo and the other one to NR. The daily NR dose was gradually escalated by 250 mg/week to the full dose of 1000 mg/day, continuing until 5 months (Fig. 1A). The primary endpoint was the change in muscle and WAT mitochondrial biogenesis (clinicaltrials.gov entry NCT03951285). For the list of secondary endpoints, see https://clinicaltrials.gov/ct2/show/NCT03951285?term=nicotinamide+riboside&draw=4&rank=29. Figure 1A and fig. S1 present the study design and the procedures for the selection of study subjects and data analyses, respectively.

**Table 1.** Baseline characteristics of the twins from the BMI-discordant and BMI-concordant pairs. Data are shown as means ± SD or median (interquartile range). *P* values were obtained using paired Wilcoxon signed-rank test to examine the differences on anthropometric and clinical parameters between the cotwins at baseline in the twins from the BMI-discordant (*n* = 16 twin pairs/32 individuals) and concordant (*n* = 4 twin pairs/8 individuals) pairs. au, arbitrary units; ND, not determined; HbA1c, hemoglobin A1c; HOMA, homeostasis model assessment; AUC, area under the curve; OGTT, oral glucose tolerance test; HDL, high-density lipoprotein; LDL, low-density lipoprotein; FFA, free fatty acids; BP, blood pressure; AST, aspartate transaminase; MCV, mean corpuscular volume; MCH, mean corpuscular hemoglobin; MCHC, mean corpuscular hemoglobin concentration; fP, fasting plasma; Fe, iron.

Variable (unit)	Discordant All (*n* = 32 individuals)	Leaner (*n* = 16 individuals)	Heavier (*n* = 16 individuals)	*P*	Concordant All (*n* = 8 individuals)	Concordant Placebo (*n* = 4 individuals)	Concordant Treated (*n* = 4 individuals)	*P*
Age (years)	39.7 (33.1–41.1)	39.7 (33.1–41.1)	39.7 (33.1–41.1)	–	40.5 (37.8–41.3)	40.5 (37.8–41.3)	40.5 (37.8–41.3)	–
Sex (female, %)	44	44	44	–	50	50	50	–
Height (cm)	173.4 ± 10.9	173.5 ± 11.5	173.3 ± 10.7	0.477	167.4 ± 10.1	167.4 ± 11.1	167.4 ± 10.8	0.875
BMI (kg/m^2^)	30.1 ± 5.7	27.4 ± 4.2	32.8 ± 5.8	<0.001	31.7 ± 6.4	31.5 ± 6.8	32.0 ± 6.9	0.269
Weight (kg)	90.7 ± 19.8	82.7 ± 17.2	98.6 ± 19.5	<0.001	88.1 ± 13.5	87.2 ± 12.2	89.1 ± 16.6	0.625
Waist-hip ratio	0.96 ± 0.09	0.9 ± 0.1	1.0 ± 0.1	<0.001	0.94 ± 0.04	0.9 ± 0.0	1.0 ± 0.0	0.250
Body fat (%)	37.1 ± 8.2	34.0 ± 7.8	40.2 ± 7.6	<0.001	33.4 ± 12.8	34.1 ± 13.8	33.1 ± 14.8	1.000
Visceral adipose tissue (cm^3^)	2155 ± 878	1754 ± 786	2556 ± 799	0.001	2117 ± 693	2017 ± 497	2217 ± 921	0.875
Subcutaneous adipose tissue (cm^3^)	6461 ± 3001	5325 ± 2442	7597 ± 3155	<0.001	7146 ± 4461	7297 ± 4799	6996 ± 4831	0.250
Adipocyte cell number (per billion)	301 (194–505)	288 (173–383)	357 (203–581)	0.010	191 (173–267)	233 (197–308)	168 (147–204)	0.125
Adipocyte cell diameter (μM)	93 ± 11	88 ± 10	97 ± 8.9	0.002	93 ± 12	89 ± 11	96 ± 12	0.250
Adipocyte cell volume (pl)	598 ± 201	508 ± 213	689 ± 145	0.007	559 ± 186	500 ± 154	617 ± 219	0.250
Adipocyte cell weight (ng)	583 (429–641)	435 (306–572)	627 (583–740)	0.007	452 (365–674)	407 (364–500)	584 (427–722)	0.250
*PPAR* γ , adipose tissue (au)	0.95 ± 0.30	1.00 ± 0.34	0.91 ± 0.25	0.569	ND	ND	ND	ND
Liver fat (%)	1.7 (0.8–4.6)	1.2 (0.5–2.0)	2.3 (1.4–9.2)	0.010	3.4 (1.2–8.7)	5.2 (0.88–9.9)	3.4 (2.0–5.4)	0.625
Lean tissue mass (kg)	54.0 ± 10.2	52.0 ± 9.6	56.1 ± 10.5	<0.001	57.5 ± 4.9	56.2 ± 5.5	58.8 ± 4.9	0.250
Total bone mass (kg)	2.9 ± 0.6	2.8 ± 0.6	2.9 ± 0.6	<0.001	3.2 ± 0.5	3.2 ± 0.6	3.3 ± 0.5	0.500
Basal metabolic rate (kcal/day)	1874 ± 382	1781 ± 370	1968 ± 381	<0.001	1798 ± 262	1782 ± 265	1813 ± 298	0.625
Caloric intake (kcal/day)	2297 ± 441	2244 ± 434	2349 ± 455	0.463	2003 ± 655	1872 ± 419	2134 ± 882	1.000
Protein intake (g)	96.2 (81.6–115)	95.6 (84.5–115.3)	96.3 (79.2–116.4)	0.900	81.8 (65.3–103.5)	81.5 (6.2–100.2)	84.0 (65.7–110.1)	0.625
Fat intake (g)	103.6 ± 28.5	97.7 ± 26.0	109.6 ± 30.4	0.562	78.4 ± 29.7	69.8 ± 17.5	87.1 ± 39.5	0.875
Carbohydrate intake (g)	211.5 ± 51.1	216.0 ± 50.0	207.0 ± 53.4	0.744	212.4 ± 68.2	234.9 ± 55.1	221.3 ± 93.2	0.875
Niacin equivalent (mg/day)	44.5 (36.1–49.9)	45.4 (36.7–49.3)	43.9 (33.5–52.9)	0.706	33.0 (31.0–40.7)	32.4 (30.6–36.5)	35.8 (31.9–41.6)	0.875
Physical activity (total Baecke)	7.8 ± 1.5	8.0 ± 1.7	7.7 ± 1.4	0.802	8.4 ± 1.4	8.1 ± 0.7	8.7 ± 1.9	0.750
Alcohol consumption (doses/week)	1.0 (0.0–2.0)	0.9 (0–3.8)	5.0 (1.8–9.3)	0.009	1.0 (0.0–2.9)	1.0 (0.5–1.4)	2.0 (0.0–4.6)	0.371
Current smoker (number)	7	4	3	–	3	2	1	–
HbA1c (mmol/mol)	34 (32–36)	34 (32–35)	35 (34–36)	0.037	36 (33–37)	35 (33–36)	37 (35–38)	1.000
Fasting glucose (mM)	5.6 (5.4–5.8)	5.6 (5.3–5.7)	5.7 (5.4–5.8)	0.157	5.8 (5.1–5.8)	5.4 (5.0–5.8)	5.8 (5.6–5.8)	0.375
Fasting insulin (mlU/liter)	6.0 (4.5–9.8)	4.7 (3.8–6.7)	7.9 (5.3–12.5)	0.018	8.0 (3.9–12.9)	7.7 (3.9–11.5)	9.7 (4.1–14.9)	0.375
Fasting C-peptide (nM)	0.51 (0.39–0.65)	0.47 (0.39–0.60)	0.56 (0.43–0.83)	0.018	0.69 (0.44–1.1)	0.69 (0.41–0.98)	0.80 (0.44–1.2)	0.125
HOMA index	1.5 (1.0–2.1)	1.1 (0.94–1.6)	2.0 (1.3–3.2)	0.022	1.9 (0.96–3.4)	1.8 (0.96–2.7)	2.5 (1.0–3.8)	0.250
Matsuda index	5.7 ± 2.5	6.8 ± 2.4	4.7 ± 2.1	<0.001	8.2 ± 6.2	8.8 ± 6.9	7.5 ± 6.5	0.125
Glucose AUC during OGTT	15.7 ± 2.5	15.2 ± 2.5	16.2 ± 2.4	0.042	15.8 ± 3.6	14.8 ± 4.1	16.8 ± 3.3	0.125
Insulin AUC during OGTT	90 (72–135)	85.3 (67.7–116.9)	96.6 (78.4–175.2)	0.006	95.5 (30.9–188.0)	90.0 (30.3–161.3)	115.2 (39.2–188.0)	0.625
Adiponectin (μg/ml)	3.2 (2.1–4.3)	3.2 (2.7–3.8)	3.1 (1.6–5.2)	0.454	3.4 (2.2–8.5)	3.4 (2.8–6.8)	5.2 (3.6–6.9)	0.500
Cholesterol (total, mM)	4.7 ± 0.8	4.7 ± 0.7	4.7 ± 0.9	0.660	4.8 ± 0.6	4.7 ± 0.5	4.8 ± 0.8	0.875
HDL (mM)	1.3 (1.2–1.6)	1.4 (1.2–1.5)	1.3 (1.1–1.7)	0.224	1.3 (1.2–1.5)	1.3 (1.2–1.4)	1.4 (1.2–1.6)	0.625
LDL (mM)	3.0 ± 0.77	3.0 ± 0.71	3.1 ± 0.83	0.196	3.2 ± 0.41	3.1 ± 0.41	3.3 ± 0.45	0.586
Triglycerides (mM)	0.95 (0.71–1.2)	0.81 (0.61–1.0)	1.0 (0.73–1.3)	0.118	1.0 (0.70–1.1)	1.0 (0.88–1.0)	0.92 (0.70–1.2)	0.625
FFA (μM)	444 (376–551)	415 (362–500)	510 (428–700)	0.542	398 (332–527)	428 (325–531)	394 (332–482)	0.625
Systolic BP (mmHg)	133 ± 22	131 ± 24	135 ± 22	0.141	120 ± 12	114 ± 12	126 ± 7.8	0.098
Diastolic BP (mmHg)	83 ± 12	83 ± 12	84 ± 11	0.706	77 ± 8	77 ± 7.7	80 ± 5.9	0.197
Pulse (per min)	66.9 ± 10.7	64.5 ± 9.2	69.3 ± 11.8	0.084	61.9 ± 6.4	63.0 ± 8.0	60.8 ± 5.1	0.461
Hs-CRP (mg/liter)	1.9 (0.67–3.9)	1.7 (0.61–3.0)	2.3 (0.73–5.8)	0.117	1.3 (0.83–5.4)	1.6 (1.3–10.7)	0.99 (0.55–3.3)	0.250
Total homocysteine (μM)	11.0 (9.30–12.0)	10.0 (9.55–12.0)	11.0 (9.28–12.8)	0.500	13.0 (11.5–13.8)	13.0 (12.2–13.8)	12.5 (11.5–15.0)	1.000
ALT (U/liter)	22.0 (17.8–42.8)	20.0 (16.5–33.5)	33.0 (19.5–49.0)	0.012	22.5 (19.8–32.0)	23.5 (18.3–38.8)	22.5 (21.5–28.3)	0.581
AST (U/liter)	24.0 (21.8–31.0)	24.0 (19.8–29.2)	24.0 (22.8–31.0)	0.139	26.5 (24.0–29.5)	26.5 (24.8–30.0)	26.5 (24.0–29.5)	0.586
Creatinine (μM)	73.7 ± 12.9	73.2 ± 13.6	74.3 ± 12.7	0.569	76.6 ± 12.0	74.0 ± 13.2	79.3 ± 12.1	0.125
Hemoglobin (g/liter)	142 ± 9	142 ± 8	143 ± 9	0.659	136 ± 11	137 ± 10	136 ± 13	0.625
Hematocrit (%)	42.0 ± 2.2	41.9 ± 2.1	42.1 ± 2.4	0.680	40.6 ± 3.2	40.8 ± 2.9	40.5 ± 3.9	0.850
Erythrocytes (×10^12^/liter)	4.8 ± 0.3	4.8 ± 0.37	4.8 ± 0.31	0.103	4.5 ± 0.4	4.5 ± 0.4	4.6 ± 0.5	0.875
MCV (fl)	87.9 ± 3.5	88.4 ± 4.2	87.4 ± 2.6	0.162	89.0 ± 3.3	90.3 ± 2.1	87.8 ± 4.2	0.098
MCH (pg)	29.8 ± 1.3	29.9 ± 1.5	29.6 ± 1.2	0.182	30.1 ± 1.4	30.5 ± 1.0	29.8 ± 1.7	0.371
MCHC (g/liter)	338 (334–342)	338 (334–342)	337 (335–342)	0.609	338 (335–335)	338 (336–340)	339 (335–343)	0.345
Leukocytes (×10^9^/liter)	6.4 ± 1.5	6.5 ± 1.8	6.3 ± 1.2	0.816	6.7 ± 2.3	7.3 ± 3.0	6.2 ± 1.7	0.423
Thrombocytes (×10^9^/liter)	243 ± 55	244 ± 60	241 ± 52	0.860	247 ± 42	234 ± 29	260 ± 53	0.125
fP-Fe (μM)	19.0 ± 6.9	19.8 ± 7.6	18.1 ± 6.3	0.469	20.0 ± 7.3	20.0 ± 6.4	20.0 ± 9.1	0.875

On average, both the BMI-discordant and BMI-concordant twin groups crossed the cutoff for obesity (BMI mean 30.1 ± SD 5.7 and 31.7 ± 6.4, respectively). The heavier cotwins of the BMI-discordant twin pairs (BMI 32.8 ± 5.8) had expectedly higher measures of adiposity, insulin resistance, basal metabolic rate, liver fat, and enzyme alanine aminotransferase (ALT) than their leaner cotwins (BMI 27.4 ± 4.2) (Table 1). The total intake of food or macronutrients or physical activity did not differ between the leaner and the heavier cotwins of the BMI-discordant pairs, but the heavier cotwins consumed more alcohol (Table 1). Both twins from the BMI-concordant pairs randomly assigned to receive either placebo (BMI 31.5 ± 6.8) or NR (BMI 32.0 ± 6.9) appeared very similar in their baseline characteristics (Table 1). Overall, most of the clinical values were within reference values in both twin-pair groups (Table 1).

### NR increases whole-blood NAD^+^ concentrations and tissue NAD^+^ biosynthesis

To elucidate the compliance and the effect of NR on NAD^+^ metabolism in the twins from the BMI-discordant pairs, we analyzed NAD metabolome by targeted liquid chromatography–mass spectrometry on the whole-blood samples. At baseline, blood NAD metabolites did not differ between the leaner and the heavier cotwins (fig. S2, A to F). NR boosted whole-blood NAD^+^ levels by 2.3-fold (Fig. 1B) in all twins from the BMI-discordant pairs. In line, nicotinic acid adenine dinucleotide (NAAD), the validated biomarker for NR supplementation and an enhanced rate of NAD^+^ synthesis (*14*), was elevated upon NR (Fig. 1C). NR also significantly increased the levels of nicotinamide mononucleotide (NMN), the phosphorylated form of NR (Fig. 1D), and nicotinic acid riboside (NAR), the deamidated form of NR (Fig. 1E). *N*-methyl-4-pyridone-5-carboxamide (Me4py) was elevated by eightfold (Fig. 1F), suggesting an enhanced elimination of NR’s degradation product NAM via methylation. Of the other NAD metabolites, nicotinamide adenine dinucleotide phosphate (NADP) trended to decrease (Fig. 1G), while adenosine diphosphate ribose (ADPR) did not significantly change (Fig. 1H). The heavier and the leaner cotwins were similar in their response to NR (the delta changes, i.e., the changes from baseline to 5 months), except that the increase in Me4py levels was more pronounced in the heavier cotwins compared with the leaner cotwins (Table 2). Overall, NR was effectively metabolized, and it had a great potency to increase blood NAD metabolites.

**Table 2.** The delta response (the change from baseline to 5 months) to NR in the twins from the BMI-discordant pairs. Data are shown as means ± SD (normally distributed variables) or median (interquartile range, for skewed variables). *P* values were obtained using paired Wilcoxon signed-rank test to examine the differences on delta change (the change from baseline to 5 months) between the leaner and the heavier cotwins from the BMI-discordant pairs (*n* = 16 twin pairs/32 individuals). *16S*, 16*S* rRNA; *CYTB*, cytochrome b; *DLOOP*, D-loop region; *NDUFB8*, NADH:ubiquinone oxidoreductase subunit B8; *MT-ND5*, mitochondrially encoded NADH dehydrogenase 5; *SDHB*, succinate dehydrogenase complex iron sulfur subunit B; *UQCRC2*, ubiquinol–cytochrome c reductase core protein 2; *MT-CO1*, mitochondrially encoded cytochrome c oxidase 1*; CLPP*, caseinolytic mitochondrial matrix peptidase proteolytic subunit; *HSPD1*, heat shock protein family D (Hsp60) member 1; *CPT1*β, carnitine palmitoyltransferase 1B; *PAX3*, paired box 3; *MYF5*, myogenic factor 5.

	Leaner Δ (*n* = 3-16 individuals)	Heavier Δ (*n* = 3-16 individuals)	*P*
NAD metabolites			
NAD^+^, whole blood (μM)	30.1 ± 20.0	33.4 ± 12.1	0.519
NAAD, whole blood (μM)	0.15 (0.08–0.34)	0.26 (0.11–0.31)	0.533
NMN, whole blood (μM)	0.12 ± 0.15	0.23 ± 0.08	0.139
NAR, whole blood (μM)	0.0 (0.0–0.0)	0.0 (0.0–0.0)	1.000
Me4py, whole blood (μM)	1.6 ± 0.59	1.9 ± 0.46	0.014
NADP, whole blood (μM)	−0.82 (−1.7 to −0.69)	−0.15 (−0.88 to 0.60)	0.175
ADPR, whole blood (μM)	−0.85 ± 3.1	0.42 ± 2.8	0.557
Expression of NAD^+^ biosynthesis genes			
*NRK1*, muscle (au)	0.08 ± 0.14	0.04 ± 0.13	0.424
*NAMPT*, muscle (au)	0.13 (−0.04 to 0.29)	0.19 (0.09–0.18)	0.742
*NMNAT3*, muscle (au)	−0.24 ± 0.27	−0.06 ± 0.26	0.129
*NAPRT*, muscle (au)	−0.10 ± 0.20	−0.10 ± 0.21	1.000
*NRK1*, adipose tissue (au)	0.01 ± 0.28	0.19 ± 0.23	0.068
*NAMPT*, adipose tissue (au)	0.06 (−0.08 to 0.23)	−0.04 (−0.13 to 0.20)	0.922
*NMNAT3*, adipose tissue (au)	0.01 (−0.25 to 0.24)	0.09 (0.04–0.23)	0.110
*NAPRT*, adipose tissue (au)	−0.05 (−0.24 to 0.12)	−0.15 (−0.50 to −0.05)	0.938
Anthropometric and body composition			
BMI (kg/m^2^)	0.89 ± 1.5	1.0 ± 1.2	0.224
Weight (kg)	2.7 ± 4.3	2.6 ± 3.4	0.339
Body fat (%)	1.5 ± 2.6	0.62 ± 2.2	0.404
Visceral adipose tissue (cm^3^)	250 ± 307	101 ± 560	0.610
Subcutaneous adipose tissue (cm^3^)	683 ± 1240	−46 ± 1295	1.000
Adipose tissue (cm^3^)	933 ± 1476	55 ± 1760	1.000
Adipocyte cell number (per billion)	−123 (−150 to −4.0)	−6.2 (−337 to 95)	0.599
Adipocyte cell diameter (μM)	4.5 ± 16	1.0 ± 17	0.454
Adipocyte cell volume (pl)	111 ± 356	110 ± 17	0.639
Adipocyte cell weight (ng)	−6.3 (−113 to 186)	38 (−159 to 365)	0.639
*PPAR*γ, adipose tissue (au)	0.13 ± 0.26	0.15 ± 0.22	0.765
Waist-hip ratio	0.02 ± 0.05	0.00 ± 0.03	0.211
Liver fat (%)	0.05 (−0.25 to 0.55)	−0.10 (−0.70 to 1.10)	0.635
Lean tissue mass (g)	158 ± 1234	747 ± 1322	0.193
Total bone mass (g)	10.5 ± 32.2	6.6 ± 17.4	0.940
Basal metabolic rate (kcal/day)	30.8 ± 54.0	47.0 ± 44.4	0.313
Caloric intake (kcal/day)	20 ± 627	−135 ± 687	0.562
Niacin equivalent (mg/day)	−4.0 (−12.5 to 2.1)	0.47 (−14.7 to 11.2)	0.528
Physical activity (total Baecke)	0.0 ± 1.5	−0.3 ± 0.6	0.401
Alcohol intake (doses/week)	0.49 (0.0–0.94)	−0.19 (−0.50 to 0.13)	0.074
Glucose homeostasis			
HbA1c (mmol/mol)	1.0 (0.0–1.0)	0.0 (−2.0 to 1.5)	0.037
Fasting glucose (mM)	0.10 (−0.10 to 0.20)	0.20 (−0.10 to 0.33)	0.089
Fasting insulin (mlU/liter)	1.2 (−0.1 to 4.1)	1.5 (−1.2 to 3.6)	0.670
Fasting C-peptide (nM)	0.05 (0.01–0.15)	0.11 (−0.0 to 0.18)	0.599
HOMA index	0.35 (−0.11 to 1.0)	0.37 (−0.22 to 0.98)	0.804
Matsuda index	−1.4 ± 2.1	−1.0 ± 1.8	0.305
Glucose AUC during OGTT	0.3 ± 2.2	0.6 ± 1.4	0.635
Insulin AUC during OGTT	14.8 (−8.8 to 30.7)	11.0 (−1.6 to 64.8)	0.635
Adiponectin (ng/ml)	17 (−111 to 516)	−105 (−796 to 364)	0.151
Cardiovascular health			
Cholesterol (total, mM)	0.03 ± 0.33	0.07 ± 0.32	0.889
HDL (mM)	−0.08 (−0.17 to 0.03)	−0.05 (−0.18 to 0.08)	0.231
LDL (mM)	0.03 ± 0.35	−0.08 ± 0.59	0.551
Triglycerides (mM)	0.13 (−0.11 to 0.26)	0.15 (−0.08 to 0.27)	0.231
FFA (μM)	−9.48 (−89.3 to 47.7)	36.5 (−154 to 66.5)	0.542
Systolic BP (mmHg)	−4.0 ± 17.5	−1.7 ± 17.5	0.615
Diastolic BP (mmHg)	−0.5 ± 9.5	1.1 ± 13.9	0.624
Pulse (per min)	2.1 ± 9.1	0.14 ± 12.9	0.575
Hs-CRP (mg/liter)	0.13 (−0.39 to 0.65)	−0.07 (−0.43 to 0.71)	0.391
Total homocysteine (μM)	2.0 (0.15–2.5)	0.90 (−0.93 to 1.2)	0.024
Mitochondrial parameters			
Mitochondrial number, muscle (number/muscle fiber area/10 μm^2^)	12.6 ± 23.0	12.0 ± 29.9	1.000
Mitochondrial area, muscle (relative area %)	0.67 (0.01–1.12)	0.91 (−0.33 to 2.2)	1.000
Mitochondrial perimeter (μm)	−0.02 ± 0.28	0.04 ± 0.35	0.966
Mitochondrial diameter (μm)	0.01 (−0.02 to 0.02)	−0.02 (−0.02 to 0.02)	0.413
Mitochondrial form factor	−0.09 (−0.17 to 0.25)	0.07 (−0.06 to 0.27)	0.365
Mitochondrial aspect ratio	−0.05 (−0.25 to 0.26)	0.09 (−0.08 to 0.24)	0.240
*16S*, muscle (au)	0.27 (0.25–0.53)	0.30 (0.01–0.68)	0.846
*CYTB*, muscle (au)	0.31 (0.16–0.37)	0.18 (0.11–0.47)	0.622
*DLOOP*, muscle (au)	0.29 ± 0.16	0.31 ± 0.24	0.922
*16S*, adipose tissue (au)	−0.09 ± 0.42	−0.19 ± 0.40	0.542
*CYTB*, adipose tissue (au)	−0.06 (−0.52 to 0.06)	−0.04 (−0.34 to 0.08)	0.635
*DLOOP*, adipose tissue (au)	−0.11 ± 0.41	−0.11 ± 0.34	0.934
*SIRT1,* muscle (au)	0.15 (−0.27 to 0.32)	0.48 (0.27–0.73)	0.064
*SIRT3,* muscle (au)	−0.28 (−0.94 to 0.25)	0.15 (−0.09 to 0.23)	0.054
*ERR*α, muscle (au)	0.15 ± 0.23	0.15 ± 0.19	0.413
*NRF1*, muscle (au)	−0.08 ± 0.25	−0.16 ± 0.32	0.547
*PGC1*α, muscle (au)	−0.07 ± 0.35	−0.04 ± 0.38	0.791
*TFAM*, muscle (au)	0.16 ± 0.34	0.28 ± 0.36	0.244
*MFN2*, muscle (au)	0.14 ± 0.15	0.06 ± 0.21	0.520
*NDUFB8*, muscle (au)	−0.09 ± 0.25	−0.19 ± 0.27	0.770
*MT-ND5*, muscle (au)	−0.10 (−0.59 to 0.20)	−0.17 (−0.44 to 0.01)	0.893
*SDHB*, muscle (au)	−0.04 ± 0.24	0.12 ± 0.55	0.734
*UQCRC2*, muscle (au)	0.01 ± 0.19	0.02 ± 0.23	0.685
*COX4,* muscle (au)	−0.07 (−0.18 to 0.16)	0.05 (−0.14 to 0.26)	0.938
*MT-CO1*, muscle (au)	−0.06 ± 0.31	−0.02 ± 0.42	0.820
*ATP5A*, muscle (au)	0.10 ± 0.22	0.14 ± 0.16	0.910
*CLPP*, muscle (au)	−0.01 ± 0.17	0.04 ± 0.19	0.557
*HSPD1*, muscle (au)	−0.04 ± 0.26	0.00 ± 0.25	0.733
*CPT1*β, muscle (au)	−0.15 ± 0.63	−0.03 ± 0.73	0.765
*ERR*α, adipose tissue (au)	−0.07 ± 0.46	−0.02 ± 0.27	0.339
*NRF1*, adipose tissue (au)	0.08 (−0.03 to 0.16)	0.11 (−0.09 to 0.21)	0.787
*TFAM*, adipose tissue (au)	−0.02 ± 0.39	−0.05 ± 0.26	1.000
*MFN2*, adipose tissue (au)	−0.07 ± 0.21	−0.05 ± 0.25	0.898
*NDUFB8*, adipose tissue (au)	−0.30 (−0.52 to −0.07)	−0.36 (−0.42 to −0.11)	0.588
*MT-ND5*, adipose tissue (au)	−0.19 (−0.38 to 0.02)	−0.17 (−0.43 to 0.01)	0.622
*SDHB*, adipose tissue (au)	−0.05 (−0.25 to 0.09)	−0.07 (−0.13 to 0.11)	0.742
*UQCRC2*, adipose tissue (au)	0.30 ± 0.38	0.16 ± 0.26	0.426
*COX4*, adipose tissue (au)	0.04 (−0.39 to 0.30)	−0.01 (−0.15 to 0.11)	1.000
*MT-CO1*, adipose tissue (au)	0.02 (−0.14 to 0.11)	−0.13 (−0.30 to −0.17)	0.734
*ATP5A*, adipose tissue (au)	−0.04 ± 0.32	0.06 ± 0.23	0.365
*CLPP*, adipose tissue (au)	−0.05 (−0.28 to 0.08)	−0.02 (−0.13 to 0.08)	0.359
*HSPD1*, adipose tissue (au)	0.03 (−0.05 to 0.14)	−0.01 (−0.07 to 0.17)	0.320
Satellite cells			
*PAX7*, muscle (au)	−0.31 ± 0.30	−0.26 ± 0.45	0.688
PAX7^+^ cells (PAX7^+^ cells/muscle fiber area)	−6.4 ± 3.6	−3.9 ± 2.6	0.578
*PAX7/MYOG* (ratio)	−0.63 ± 0.75	−1.1 ± 0.22	0.500
*PAX3/MYOG* (ratio)	−934 (−5960 to −603)	−2420 (−5370 to −1270)	0.750
*MYF5/MYOD* (ratio)	−0.03 (−0.52 to 0.32)	0.19 (0.01–1.52)	0.500
*MYMK/MYOD* (ratio)	1000 (685–2340)	−319 (−508 to 1630)	0.250

To investigate the influence of NR on tissue NAD^+^ biosynthesis, we measured mRNA expression of NAD^+^ biosynthetic enzymes from muscle and WAT. At baseline, in comparison to the leaner cotwins of the BMI-discordant pairs, the heavier cotwins exhibited significantly lower expression of NMN adenylyltransferase 3 (*NMNAT3*), one of the *NAMN*/*NMN* adenylyltransferase isoforms, in muscle and WAT (fig. S2, G and H). The expression of the NR-metabolizing enzyme NR kinase 1 (*NRK1*) was also lower in WAT of the heavier cotwins (fig. S2H). In the twins from the BMI-discordant pairs, NR increased the muscle expression of NAM phosphoribosyltransferase (*NAMPT*), the enzyme converting NAM toward NAD^+^, and trended to up-regulate *NRK1* while decreasing *NMNAT3* (Fig. 1I). In WAT, *NRK1* expression was significantly elevated, while *NAMPT* and *NMNAT3* were unchanged upon NR (Fig. 1J). NR down-regulated the Preiss-Handler pathway enzyme nicotinic acid phosphoribosyltransferase (*NAPRT*) in both muscle and WAT (Fig. 1, I and J), indicating decreased niacin utilization and/or increased reliance on the salvage pathway enzymes upon NR. The NR-induced alterations in the mRNA expression of NAD^+^ biosynthetic enzymes were similar in both cotwins, except that NR tended to increase WAT *NRK1* expression more in the heavier cotwins compared with the leaner cotwins (Table 2). As a whole, NR promoted both muscle and WAT NAD^+^ biosynthesis via the salvage pathways.

### Body weight and fat percentage increased during the study

We next determined the impact of NR on body composition in the twins from the BMI-discordant pairs. During the study, body weight (~3 kg) and the whole-body fat percentage increased significantly, and the size of all WAT depots tended to elevate in twins from the BMI-discordant pairs (Table 3). There were no significant changes in adipocyte number, diameter, volume, and weight upon NR (Table 3). However, the expression of peroxisome proliferator–activated receptor γ (*PPAR*γ), the essential transcription factor controlling adipogenesis, was up-regulated after NR supplementation (Table 3). Lean tissue (muscle) and bone mass and liver fat content remained unaltered (Table 3). Basal metabolic rate was significantly enhanced probably due to the increase in body weight (Table 3). Food diaries and questionnaires showed no marked changes in the intake of food, macronutrients, niacin equivalent, alcohol, or physical activity (Table 3). The changes in the analyzed parameters from baseline to 5 months did not differ between the leaner and the heavier cotwins (Table 2). Together, body weight and fat percentage increased during the NR intervention, but we did not observe clear changes in the self-reported lifestyle measures.

**Table 3.** The effect of NR on body composition and lifestyle factors in the twins from the BMI-discordant pairs. Data are shown as means ± SD (normally distributed variables), median (interquartile range, for skewed variables), or proportion (%, for categorical variables). *P* values were obtained using paired Wilcoxon signed-rank test to examine the effect of NR supplementation on anthropometric and clinical parameters in the twins from the BMI-discordant pairs (*n* = 16 twin pairs/32 individuals).

Variable (unit)	All Pre (*n* = 32 individuals)	All Post (*n* = 32 individuals)	*P*	Leaner Pre (*n* = 16 individuals)	Leaner Post (*n* = 16 individuals)	*P*	Heavier Pre (*n* = 16 individuals)	Heavier Post (*n* = 16 individuals)	*P*
BMI (kg/m^2^)	30.1 ± 5.7	31.1 ± 6.1	0.001	27.4 ± 4.2	28.3 ± 4.9	0.038	32.8 ± 5.8	33.8 ± 6.1	0.007
Weight (kg)	90.7 ± 19.8	93.3 ± 20.4	<0.001	82.7 ± 17.2	85.4 ± 18.6	0.010	98.6 ± 19.5	101.2 ± 19.6	0.010
Waist-hip ratio	0.96 ± 0.09	0.97 ± 0.09	0.172	0.94 ± 0.08	0.96 ± 0.08	0.065	0.98 ± 0.09	0.98 ± 0.10	0.918
Body fat (%)	37.1 ± 8.2	38.2 ± 8.2	0.008	34.0 ± 7.8	35.5 ± 8.1	0.016	40.2 ± 7.6	40.9 ± 7.6	0.117
Visceral adipose tissue (cm^3^)	2155 ± 878	2440 ± 926	0.014	1754 ± 785	2097 ± 724	0.014	2555 ± 799	2811 ± 1005	0.541
Subcutaneous adipose tissue (cm^3^)	6461 ± 3001	7049 ± 3027	0.061	5325 ± 2442	6179 ± 2891	0.056	7597 ± 3155	7990 ± 3001	0.610
Adipocyte cell number (per billion)	301 (194–505)	230 (140–374)	0.092	288 (173–383)	211 (128–265)	0.169	357 (203–581)	352 (202–471)	0.454
Adipocyte cell diameter (μM)	93 ± 11	95.4 ± 16.4	0.440	88 ± 10	92.4 ± 16.5	0.524	97 ± 8.9	98.4 ± 16.3	0.804
Adipocyte cell volume (pl)	598 ± 201	709 ± 381	0.328	508 ± 213	618 ± 350	0.639	689 ± 145	799 ± 401	0.489
Adipocyte cell weight (ng)	583 (429–641)	573 (379–833)	0.328	435 (306–572)	462 (358–659)	0.639	627 (583–740)	614 (459–954)	0.489
*PPAR*γ, adipose tissue (au)	0.95 ± 0.30	1.1 ± 0.23	0.025	1.00 ± 0.34	1.2 ± 0.23	0.206	0.91 ± 0.25	1.1 ± 0.23	0.092
Liver fat (%)	1.7 (0.8–4.6)	2.1 (0.6–5.4)	0.657	1.2 (0.5–2.0)	1.9 (0.4–2.4)	0.638	2.3 (1.4–9.2)	2.2 (0.7–10.6)	0.787
Lean tissue mass (kg)	54.0 ± 10.2	54.5 ± 10.5	0.197	52.0 ± 9.6	52.1 ± 9.6	0.980	56.1 ± 10.5	56.8 ± 11.1	0.083
Total bone mass (kg)	2.9 ± 0.6	2.9 ± 0.6	0.106	2.8 ± 0.6	2.8 ± 0.6	0.495	2.9 ± 0.6	2.9 ± 0.6	0.130
Basal metabolic rate (kcal/day)	1874 ± 382	1913 ± 390	<0.001	1781 ± 370	1812 ± 377	0.016	1968 ± 381	2015 ± 387	0.003
Caloric intake (kcal/day)	2297 ± 441	2240 ± 469	0.719	2244 ± 434	2265 ± 525	0.900	2350 ± 456	2215 ± 421	0.597
Protein intake (g)	96.2 (81.6–115)	87.5 (78.1–108)	0.454	95.6 (84.5–115)	86.6 (76.5–109)	0.782	96.3 (79.2–116)	88.4 (81.7–106)	0.562
Fat intake (g)	103.6 ± 28.5	98.5 ± 22.6	0.405	97.7 ± 26.0	98.3 ± 27.8	0.816	109.6 ± 30.4	98.7 ± 16.7	0.211
Carbohydrate intake (g)	211.5 ± 51.1	210.0 ± 57.9	0.789	216.0 ± 50.0	216.4 ± 54.9	0.980	207.0 ± 53.4	203.6 ± 61.8	0.597
Niacin equivalent (mg/day)	44.5 (36.1–49.9)	40.1 (35.4–49.8)	0.295	45.4 (36.7–49.3)	38.2 (30.8–50.7)	0.144	43.9 (33.5–52.9)	42.1 (37.0–49.8)	0.782
Physical activity (total Baecke)	7.8 ± 1.5	7.6 ± 1.4	0.562	8.0 ± 1.7	8.0 ± 1.5	0.552	7.7 ± 1.4	7.3 ± 1.2	0.090
Alcohol intake (doses/week)	1.0 (0.0–2.0)	2.1 (0.2–5.0)	0.345	0.9 (0.0–3.8)	2.1 (0.7–4.8)	0.147	5.0 (1.8–9.3)	2.0 (0.0–5.6)	0.551
Current smoker (number)	7	7	–	4	4	–	3	3	–

### Insulin sensitivity decreased during the study

To understand whether the increased body weight resulted in alterations in glucose homeostasis in the twins from the BMI-discordant pairs, we ran a comprehensive set of measures during an oral glucose tolerance test. Glucose, insulin, C-peptide, homeostasis model assessment, and Matsuda index indicated impairments in glucose homeostasis (although remaining within normal reference ranges) in all twins after NR supplementation (Table 4). No significant differences were observed regarding the changes from baseline to 5 months between the leaner and the heavier cotwins, except that hemoglobin A1c increased slightly more in the leaner cotwins than in the heavier cotwins (Table 2). Overall, our data suggest that insulin sensitivity decreased during the study.

**Table 4.** Glucose homeostasis in the twins from the BMI-discordant pairs upon NR supplementation. Data are shown as means ± SD (normally distributed variables) or median (interquartile range, for skewed variables). *P* values were obtained using paired Wilcoxon signed-rank test to examine the effect of NR supplementation on glucose metabolism related parameters in the twins from the BMI-discordant pairs (*n* = 16 twin pairs/32 individuals).

Variable (unit)	All Pre (*n* = 32 individuals)	All Post (*n* = 32 individuals)	*P*	Leaner Pre (*n* = 16 individuals)	Leaner Post (*n* = 16 individuals)	*P*	Heavier Pre (*n* = 16 individuals)	Heavier Post (*n* = 16 individuals)	*P*
HbA1c (mmol/mol)	34 (32–36)	35 (33–36)	0.353	34 (32–35)	35 (33–36)	0.032	35 (34–36)	35 (32–37)	0.693
Fasting glucose (mM)	5.6 (5.4–5.8)	5.7 (5.5–5.9)	0.027	5.6 (5.3–5.7)	5.7 (5.3–5.8)	0.483	5.7 (5.4–5.8)	5.8 (5.6–5.9)	0.018
Fasting insulin (mlU/liter)	6.0 (4.5–9.8)	7.7 (6.2–13.4)	0.023	4.7 (3.8–6.7)	7.0 (5.9–9.0)	0.069	7.9 (5.3–12.5)	12.1 (7.1–14.6)	0.169
Fasting C-peptide (nM)	0.51 (0.39–0.65)	0.63 (0.50–0.77)	0.002	0.47 (0.39–0.60)	0.55 (0.48–0.65)	0.022	0.56 (0.43–0.83)	0.74 (0.59–0.84)	0.036
HOMA index	1.5 (1.0–2.1)	2.0 (1.5–3.3)	0.016	1.1 (0.9–1.6)	1.7 (1.4–2.4)	0.073	2.0 (1.3–3.2)	3.0 (1.7–3.8)	0.135
Matsuda index	5.7 ± 2.5	4.4 ± 1.9	<0.001	6.8 ± 2.4	5.3 ± 2.0	0.068	4.7 ± 2.1	3.6 ± 1.4	0.042
Glucose AUC during OGTT	15.7 ± 2.5	16.1 ± 2.7	<0.001	15.2 ± 2.5	15.4 ± 2.6	0.286	16.2 ± 2.4	16.9 ± 2.8	0.208
Insulin AUC during OGTT	90 (72–135)	116 (88–160)	<0.001	85 (68–117)	112 (86–127)	0.217	97 (78–175)	154 (91–204)	0.173
Adiponectin (μg/ml)	3.2 (2.1–4.3)	3.2 (2.0–4.1)	0.946	3.2 (2.7–3.8)	3.3 (2.7–3.9)	0.495	3.1 (1.6–5.2)	2.8 (1.7–4.4)	0.277

Next, we examined whether cardiovascular health was affected by NR. Except for the small, clinically not meaningful decrease in high-density lipoprotein and triglycerides, no changes in blood lipid values were detected upon NR (table S1). Neither were there any changes in blood pressure, pulse rate, nor the inflammation marker high-sensitivity complement-reactive protein (CRP) (table S1). The NR’s effect on these clinical variables was similar in the leaner and the heavier cotwins (Table 2).

### NR boosts mitochondrial biogenesis in muscle but not in WAT

**Fig. 2.**
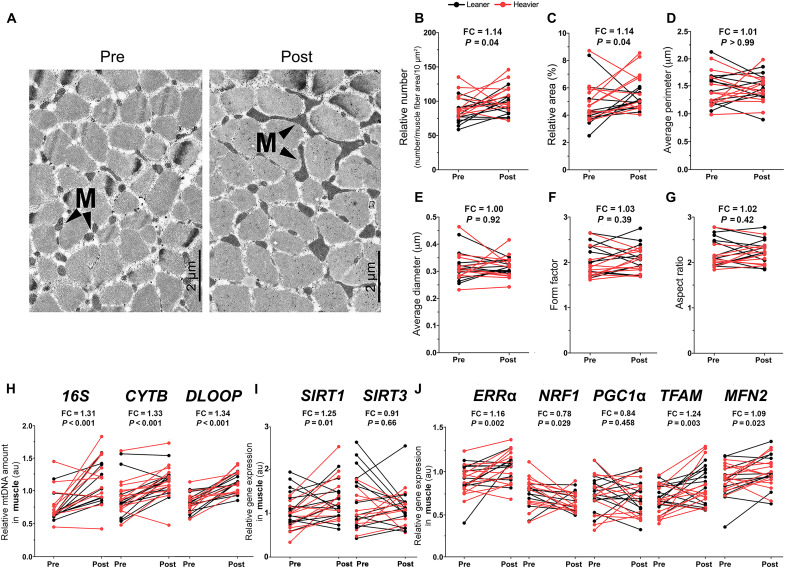
NR boosts muscle mitochondrial biogenesis in the twins from the BMI-discordant pairs. (**A**) TEM images of muscle intermyofibrillar mitochondria (M) in one representative study participant before and after NR. Magnification, ×2000. Scale bars, 2 μm. (**B** to **G**) Quantification of (B) the number of mitochondria per 10 μm^2^ relative to the muscle fiber area, (C) percentage of mitochondrial surface area per total muscle fiber area, (D) average perimeter of mitochondria, (E) average diameter of mitochondria, (F) form factor (the branching of mitochondria), and (G) aspect ratio (the length-to-width ratio of mitochondria) in muscle biopsies (*n* = 11 twin pairs/22 individuals). (**H**) Muscle relative mtDNA amount presented as a ratio of mtDNA genome per nuclear genome before versus after NR (*n* = 11 to 12 twin pairs/22 to 25 individuals). *16S*, 16*S* rRNA; *CYTB*, cytochrome b; *DLOOP*, D-loop region. (**I** and **J**) Gene expression of *SIRT1* and *SIRT3* (I) and other key regulators of mitochondrial biogenesis (J) in muscle before versus after NR (*n* = 10 to 12 twin pairs/21 to 28 individuals). Arbitrary unit indicates the relative gene expression normalized to the expression of reference genes. *SIRT3*, sirtuin 3. Lines connect the pre- and post-values of each individual, with black denoting the leaner and red denoting the heavier cotwins. Fold change indicates the mean of the post-NR value divided by the pre-NR value. *P* values were calculated using paired Wilcoxon signed-rank test. See also Table 2 and fig. S3.

Given that NR significantly elevated whole-blood and tissue NAD^+^ biosynthesis in the twins from the BMI-discordant pairs, we determined the effect of NR on mitochondrial biogenesis in these twins. Transmission electron micrographs (TEMs) of muscle samples showed that mitochondria became more abundant (~14%) in the intermyofibrillar region of type I muscle fibers upon NR (Fig. 2, A and B). In addition, mitochondria covered a larger cross-sectional area of the muscle fiber area after NR supplementation (Fig. 2C). Morphological analysis of mitochondria [perimeter, diameter, form (i.e., branching) factor, or aspect (i.e., length-to-width) ratio] did not reveal any alterations upon NR (Fig. 2, D to G). In line with TEM findings, NR clearly elevated (~30%) muscle mitochondrial DNA (mtDNA) amount (Fig. 2H) and muscle expression of the following transcription factors regulating mitochondrial biogenesis: sirtuin 1 (*SIRT1*), estrogen-related receptor α (*ERR*α*)*, transcription factor A (*TFAM*), and mitofusin 2 (*MFN2*) (Fig. 2I). In addition, NR significantly elevated the expression of muscle oxidative phosphorylation (OXPHOS) complex subunits cytochrome c oxidase subunit 4 and adenosine triphosphate synthase α (*ATP5A*) but down-regulated one complex I subunit (fig. S3, A and B). The leaner and the heavier cotwins were similar in their response to NR in terms of their muscle mitochondrial number, mtDNA amount, and gene expression levels except that the changes in the expression of *SIRT1* and sirtuin 3 tended to differ between the heavier and the leaner cotwins (Table 2). Collectively, NR markedly increased muscle biogenesis, which was associated with the up-regulation of *SIRT1*/*ERR*α/*TFAM*/*MFN2*.

We next investigated the effect of NR on WAT mitochondria in the twins from the BMI-discordant pairs. NR did not significantly influence mtDNA amount and had only a minor effect on the gene expression profile in WAT (fig. S3, C to F). The changes in these parameters did not differ between the leaner and the heavier cotwins (Table 2).

### NR promotes muscle satellite cell differentiation

**Fig. 3.**
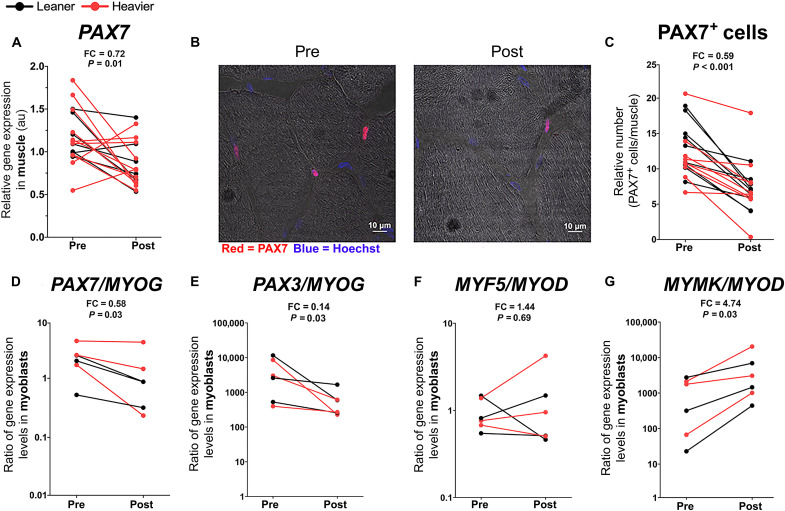
NR promotes muscle satellite cell differentiation in the twins from the BMI-discordant pairs. (**A**) Muscle gene expression level of satellite cell marker *PAX7* before versus after NR (*n* = 9 twin pairs/18 individuals). (**B**) Immunostaining of PAX7^+^ satellite cells in muscle cryosections before versus after NR in one representative study participant. PAX7 (red, satellite cells); Hoechst (blue, nuclei). Scale bars, 10 μm. (**C**) Muscle PAX7^+^ satellite cell quantification before versus after NR (*n* = 10 twin pairs/20 individuals). (**D** to **G**) Ratios of *PAX7*/*MYOG* (D), *PAX3*/*MYOG* (E), *MYF5*/*MYOD* (F), and *MYMK*/*MYOD* (G) mRNA expression in myoblasts before versus after NR (*n* = 3 twin pairs/6 individuals). *Y* axis is on a logarithmic scale. *PAX3*, paired box 3; *MYF5*, myogenic factor 5. Lines connect the pre- and post-values of each individual, with black denoting the leaner and red denoting the heavier cotwins. Fold change indicates the mean of the post-NR value divided by the pre-NR value. *P* values were calculated using paired Wilcoxon signed-rank test. See also Table 2.

As NR has been previously shown to increase the number and the function of muscle stem cells, i.e., satellite cells in mice (*10*), we investigated the effect of NR on muscle satellite cells in the twins from the BMI-discordant pairs. Muscle expression of the stem cell marker paired box 7 (*PAX7*) and the number of PAX7^+^ satellite cells were significantly reduced upon NR (Fig. 3, A to C). To further characterize the muscle stem cells, we created primary myoblast cultures from the muscle biopsies. Myoblasts derived from the NR-supplemented twins showed reduced stemness and increased differentiation, as demonstrated by a significant decrease in the expression ratio of stemness markers *PAX7* and paired box 3 over the late myogenic differentiation marker myogenin (*MYOG*) (Fig. 3, D and E). However, the expression ratio of other myogenic regulatory factors, myogenic factor 5 and myoblast determination protein 1 (*MYOD*), remained unaltered upon NR (Fig. 3F). The reduction in the muscle PAX7^+^ satellite cell number coupled with the myoblasts’ phenotypic shift toward differentiation suggested that NR may activate satellite cells and their fusion to muscle fibers (*23*). In human myoblasts, MYOD initiates the fusogenic program, for example, by up-regulating the expression of myomaker (*MYMK*), the essential myogenic fusion factor (*24*). Given that myoblasts derived from the NR-supplemented twins exhibited a significantly elevated expression ratio of *MYMK* and *MYOD* (Fig. 3G), our results suggest that NR likely drives the human myoblast fusion. NR’s effect on muscle *PAX7* expression, satellite cell number, or myoblast *PAX7*/*MYOG*, paired box 3/*MYOG*, myogenic factor 5/*MYOD*, and *MYMK*/*MYOD* ratios was similar in both cotwins (Table 2). Overall, our data indicate that NR likely facilitated the activation of satellite cells towards differentiation and fusion to the existing myofibers, resulting in the observed decline in the satellite cell number in the twins from the BMI-discordant pairs.

### NR may affect the epigenetic control of gene expression in muscle and WAT

**Fig. 4.**
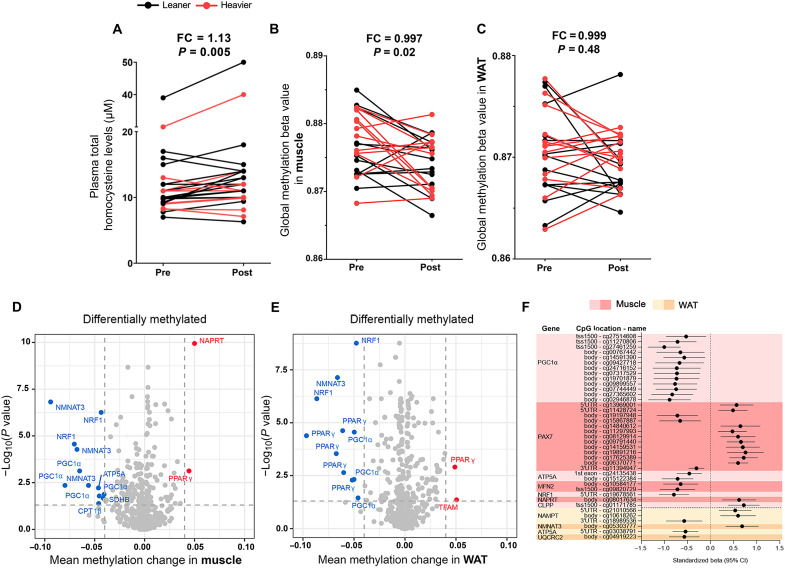
NR modifies the epigenetic control of gene expression in the twins from the BMI-discordant pairs. (**A** to **C**) Plasma total homocysteine (A) and global DNA methylation levels in (B) muscle and (C) WAT before versus after NR (*n* = 12 to 13 twin pairs/24 to 26 individuals). Lines connect the pre- and post-values of each individual, with black denoting the leaner and red denoting the heavier cotwins. Fold change indicates the mean of the post-NR value divided by the pre-NR value. (**D** and **E**) Volcano plots showing statistical significance (*y* axis) and the magnitude of the change in mean methylation beta value (*x* axis) in (D) muscle and (E) WAT upon NR (*n* = 12 to 14 twin pairs/25 to 28 individuals). Each dot represents a single CpG site (*n* = 619 and 518 in muscle and WAT, respectively). Highlighted are CpGs with FDR *P* < 0.05 (dashed horizontal line). *CPT1*β, carnitine palmitoyltransferase 1β; *SDHB*, succinate dehydrogenase complex iron sulfur subunit B. (**F**) Significant associations between CpG site methylation and gene expression in muscle (red) and WAT (yellow) upon NR (*n* = 7 to 9 twin pairs/14 to 18 individuals). Standardized beta values show the SD values in methylation changes associated with 1 SD of gene expression change. CI, confidence interval; *CLPP*, caseinolytic mitochondrial matrix peptidase proteolytic subunit; *UQCRC2*, ubiquinol–cytochrome c reductase core protein 2. *P* values for plasma total homocysteine levels were calculated using paired Wilcoxon signed-rank test. Statistical analyses of methylation data are described in Materials and Methods. See also Table 2 and tables S1 to S3.

The supplementation with NAD^+^ precursors has been suggested to induce a decline in methyl groups due to enhanced elimination of NAM via methylation (*25*). Thus, we measured the levels of circulating homocysteine, increased levels of which can be considered as a marker for compromised cellular methylation status. NR slightly but significantly increased the levels of total plasma homocysteine, especially in the leaner cotwins from the BMI discordant pairs (Fig. 4A, Table 2, and table S1), but not beyond the normal range, suggesting a reduction in the cellular methylation capacity. These findings prompted us to investigate whether NR affected tissue DNA methylation. NR significantly reduced global DNA methylation level in muscle, but not in WAT (Fig. 4, B and C), with similar effects in both cotwins (median within-pair difference of 0.001, *P* = 0.47 in muscle, and −0.001, *P* = 0.52 in WAT). Overall, our results suggest that long-term NR supplementation influences global DNA methylation in a tissue-specific manner.

Next, we performed differential DNA methylation analysis for selected genes involved in NAD^+^ biosynthesis, mitochondrial biogenesis, protein quality control, fatty acid oxidation, OXPHOS, and satellite cell identity to determine the effect of NR on muscle and WAT CpG methylation in the twins from the BMI-discordant pairs. NR altered the methylation of 173 of 619 (28%) and 210 of 518 (41%) CpG sites in muscle and WAT, respectively (Fig. 4, D and E). While NR induced both hypo- and hypermethylation, most of the analyzed CpG sites were hypomethylated (~60% in both tissues) after NR. The hypomethylated CpGs were typically located at the open sea, whereas the hypermethylated sites were mostly observed at CpG islands. Most of the hypomethylated CpGs were detected in *PPAR*γ coactivator 1α (*PGC-1*α), *NMNAT3*, and nuclear respiratory factor 1 (*NRF1*) in muscle and in *PPAR*γ and *NRF1* in WAT. The leaner and the heavier cotwins did not differ in their response to NR at their tissue CpG site methylation (table S2).

As DNA methylation may influence gene expression, we assessed associations between gene expression and CpG methylation of the respective genes. In muscle, a total of 33 statistically significant associations were observed (table S3). All 13 associations observed between *PGC-1*α expression and methylation were negative (Fig. 4F), suggesting that hypomethylation of *PGC-1*α may increase its transcription in muscle. In addition, 13 significant, mostly positive associations were detected between expression and methylation of *PAX7*. Thus, hypomethylation of *PAX7* may repress its expression in muscle. Other detected associations between gene expression and DNA methylation were represented by only up to two CpG sites at each of the genes, and the correlations were negative for *ATP5A*, *MFN2*, and *NRF1* and positive for *NAPRT* and caseinolytic mitochondrial matrix peptidase proteolytic subunit (Fig. 4F). In WAT, there were only six associations between the gene expression and CpG site methylation at the respective genes, three of which were annotated to *NAMPT* and one CpG to each of the other three genes *NMNAT3*, *ATP5A*, and ubiquinol–cytochrome c reductase core protein 2 (Fig. 4F). Although it is difficult to draw any firm conclusions on the effects of DNA methylation on gene expression, these results suggest that epigenetic control could be one of the mechanisms via which NR regulates the expression of genes participating in mitochondrial biogenesis and quality control, satellite cell stemness, NAD^+^ biosynthesis, and OXPHOS.

### NR alters plasma metabolomic profile

**Fig. 5.**
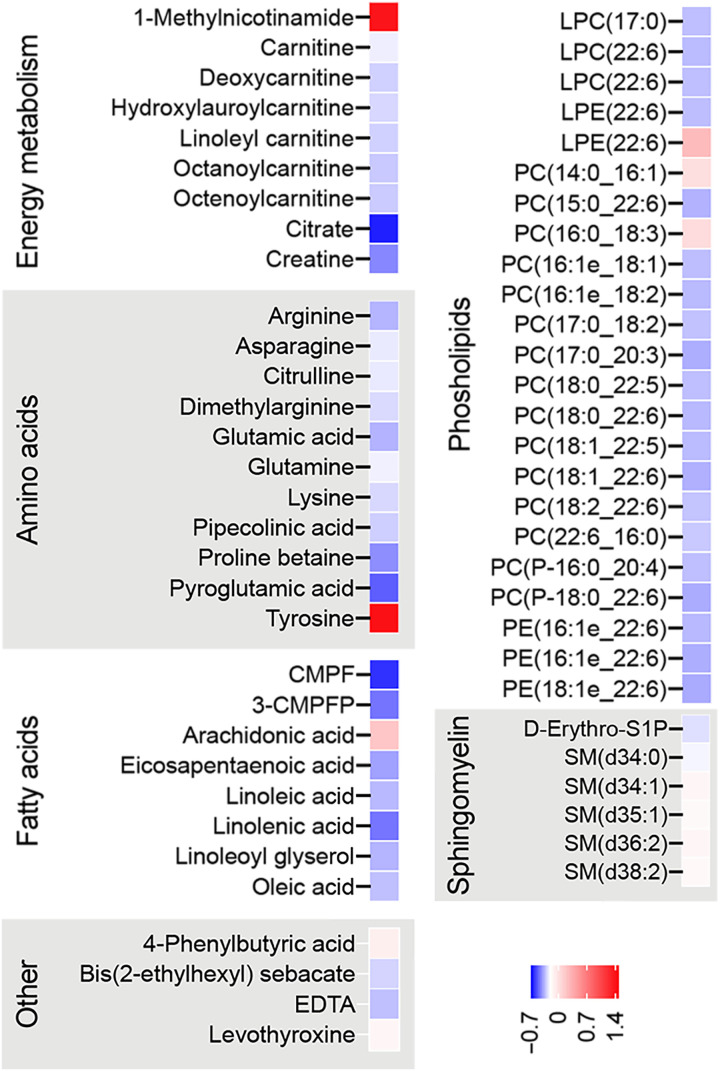
NR alters plasma metabolomic profile in the twins from the BMI-discordant pairs. Heatmaps showing significant plasma metabolite log_2_-fold changes after NR (*n* = 14 twin pairs/28 individuals; nominal *P* < 0.05). Blue color indicates lower and red color indicates higher metabolite level upon NR. CMPF, 3-carboxy-4-methyl-5-propyl-2-furanopropionic acid; 3-CMPFP, 3-carboxy-4-methyl-5-pentyl-2-furanpropanoic acid; LPC, lysophosphatidylcholine; LPE, lysophosphatidyl-ethanolamine; PC, phosphatidylcholine; PE, phosphatidylethanolamine; D-Erythro-S1P, d-erythro-sphingosine-1-phosphate; SM, sphingomyelin. Related statistical analyses are described in Materials and Methods. See also tables S4 and S5.

To understand whether NR affects the global plasma metabolomic profile, we performed a nontargeted metabolomic analysis of fasting plasma samples from the twins of the BMI-discordant pairs. With regression analysis, we found 460 significantly altered metabolites upon NR. Among these, 72 metabolites were identified with a level 1 or 2 in the Metabolomics Standard Initiative reporting standards (table S4). Of the 72 identified metabolites, the most significantly increased metabolite was methylNAM (Fig. 5), the NAM waste product, in line with the whole-blood NAD metabolome (Fig. 1F) and global DNA methylation results (Fig. 4, A and B). In addition, the levels of tyrosine, the amino acid precursor of brain catecholamines, were significantly elevated upon NR (Fig. 5). Most of the decreased metabolites fell into the following categories: energy metabolism, amino acids, fatty acids, phospholipids, and sphingomyelins. The content of metabolites central for the efficient mitochondrial energy production—carnitine, acylcarnitines, and polyunsaturated fatty acids—decreased significantly (Fig. 5), suggesting their lowered synthesis/excretion and/or increased uptake/utilization by the tissues. NR decreased the plasma levels of several amino acids including gluconeogenic amino acids (Fig. 5), indicating also their diminished synthesis/excretion and/or increased uptake/utilization by the tissues such as the liver, which is the main tissue responsible for amino acid metabolism in the postprandial state. Given that the plasma levels of 3-carboxy-4-methyl-5-propyl-2-furanopropionic acid, a putative biomarker of fatty fish intake (*26*), were significantly decreased (Fig. 5), we cannot exclude the possibility that the lowered levels of polyunsaturated fatty acids partially reflected a decline in fish intake. The decrease in phosphatidylcholines and 1,5-diaminonaphthalene was more pronounced in the heavier cotwins, but otherwise, the metabolite responses were similar in both cotwins (on the 1 or 2 metabolite identification level; table S5). Overall, our results revealed that the main plasma metabolites modulated by NR supplementation are related to mitochondrial energy metabolism, lipids, and amino acids.

### NR modulated the gut microbiota composition

**Fig. 6.**
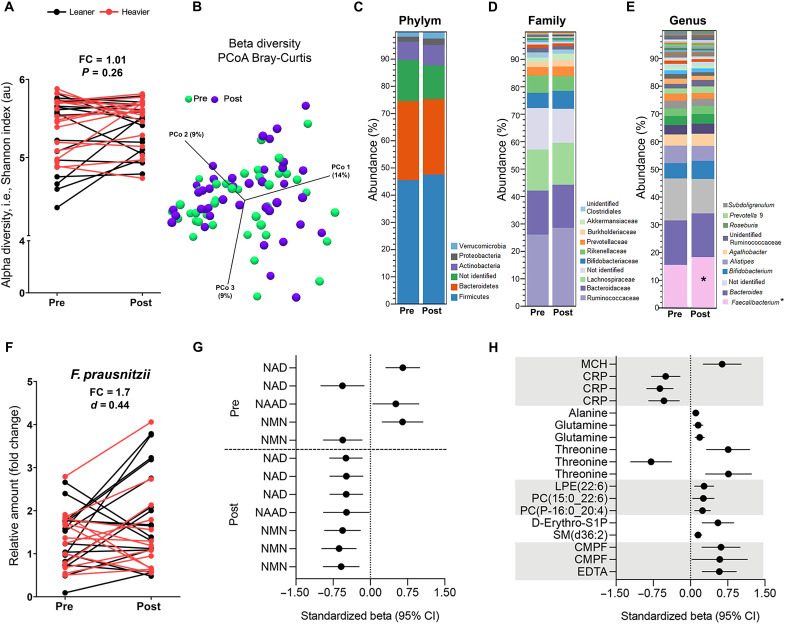
NR improves the gut microbiota composition in the twins from the BMI-discordant pairs. (**A**) Alpha-diversity of the gut microbiota before versus after NR (*n* = 11 twin pairs/22 individuals). Lines connect the pre- and post-values of each individual, with black denoting the leaner and red denoting the heavier cotwins. Fold change indicates the mean of the post-NR value divided by the pre-NR value. (**B**) Principal components analysis (PCoA) of the gut microbiota beta-diversity before versus after NR (*n* = 11 twin pairs/22 individuals). (**C** to **E**) Average gut microbiota abundance at the phylum (C), family (D), and genus (E) level before versus after NR (*n* = 11 twin pairs/22 individuals). In the legends of the bars, the phylum level shows all detected phyla in the samples and at the family and genus level. Asterisk indicates nominal *P* < 0.05. (**F**) Relative amount of *F. prausnitzii* presented as a fold change to 16*S* rRNA amount in fecal DNA before versus after NR (*n* = 11 twin pairs/22 individuals). Cohen’s *d* value of 0.44 suggests a medium effect size. (**G** and **H**) Standardized beta values showing SD values in (G) NAD metabolites associated with 1 SD in baseline abundance of *Faecalibacterium* or *Faecalibacterium* abundance change upon NR and in (H) clinical variables and plasma metabolites associated with NR-induced changes in *Faecalibacterium* (*n* = 11 twin pairs/22 individuals). Rows represent associations with *Faecalibacterium* OTUs. MCH, mean corpuscular hemoglobin. Statistical analyses are described in Materials and Methods. See also tables S6 and S7.

To evaluate whether NR modified the gut microbiota, 16*S* ribosomal RNA (rRNA) gene sequencing of fecal samples of the twins from the BMI-discordant pairs was performed. The alpha- or beta-diversity of the gut microbiota did not change during the study (Fig. 6, A and B). In contrast, NR increased the abundance of the genus *Faecalibacterium* by ~2%, but this increase did not pass the multiple testing correction via false discovery rate (FDR) (Fig. 6, C to E). As *F. prausnitzii* is the only validated species of *Faecalibacterium* genus, we quantified the NR-induced changes in *F. prausnitzii* using quantitative real-time reverse transcription polymerase chain reaction (RT-qPCR). There was an increasing ~1.7-fold effect on *F. prausnitzii* upon NR (Fig. 6F). The changes for any of the analyzed gut microbiota operational taxonomic units (OTUs) did not significantly differ between the leaner and the heavier cotwins (FDR *P* > 0.05) (table S6). Overall, our data suggest that NR increased the abundance of *F. prausnitzii*, which is one of the most important commensal bacteria of the human gut microbiota (*27*, *28*).

### The abundance of *Faecalibacterium* associates with changes in blood metabolite concentrations

As the gut microbiota can influence the response of the host to vitamin B3 supplementation (*29*), we determined associations between OTUs and blood metabolites (table S7) in the twins from the BMI-discordant pairs. Three of five *Faecalibacterium* OTU baseline levels associated positively with the change in whole-blood NAD^+^, NAAD, and NMN upon NR, suggesting that the baseline abundance of *Faecalibacterium* may support the NR-induced elevation in blood NAD metabolome (Fig. 6G). In addition, the NR supplementation–induced increase in *Faecalibacterium* abundance was negatively associated with the change in whole-blood levels of NAD^+^, NAAD, and NMN (Fig. 6G), possibly reflecting competition for NR between *Faecalibacterium* and the host. The association analysis with blood clinical variables revealed that the *Faecalibacterium* increase was negatively associated with the change in high-sensitivity CRP but positively with the change in mean corpuscular hemoglobin (Fig. 6H). Last, the *Faecalibacterium* increase was associated positively with the change in plasma gluconeogenic amino acids (alanine, glutamine, and threonine), phospholipids, and sphingomyelins (Fig. 6H). Together, our results imply that *F. prausnitzii* may contribute to the regulation of NAD^+^, inflammation, and amino acid and lipid metabolism in humans.

### NR and placebo affected adiposity and metabolic health similarly in BMI-concordant twin pairs

We lastly studied the impact of NR on whole-blood NAD metabolites, body composition, and clinical variables in comparison to placebo in four BMI-concordant twin pairs. We observed that NR treatment appeared to induce whole-blood NAD^+^, NAAD, and Me4py in comparison to placebo (fig. S2, I to N), confirming compliance to the NR treatment. Of the body composition parameters, body weight (~3 kg), fat percentage, liver fat percentage, and adipocyte weight and volume increased (however, statistically nonsignificantly probably due to small sample size) in the placebo-treated cotwins, while these variables remained stable in the NR-treated cotwins (table S8). We did not observe any changes in the lifestyle factors, glucose and lipid metabolism, and cardiovascular health–related variables in the placebo- or the NR-treated twins from the BMI-concordant pairs (table S8). In conclusion, on the basis of these findings, NR’s effect on body composition and metabolic health did not markedly differ from the effects of placebo.

### NR supplementation was well tolerated

NR supplementation was well tolerated by the study participants. Reported side effects included muscle pain, gastrointestinal irritation, sweating, nausea, and headache but not cutaneous flushing typically caused by niacin (*4*). The placebo-treated twins did not report any side effects. Safety parameters including, for example, the kidney function marker creatinine, liver enzymes, and complete blood count remained unaltered in the blood samples of all study participants (tables S8 and S9). The only exception was that there was a statistically but not clinically significant reduction in hemoglobin in the twins from the BMI-discordant pairs upon NR (table S9). However, this normocytic, normochromic condition (table S9) was likely related to blood loss due to blood sampling. As a whole, these results showed that long-term NR supplementation did not markedly alter the measured safety parameters.

## DISCUSSION

Boosting of NAD^+^ levels is currently under the focus of intensive research as a potential treatment option for metabolic diseases (*2*). In our study, NR effectively increased the levels of whole-blood NAD^+^, NAAD, NMN, and Me4py. This is in line with the previous short-term NR clinical studies (*14*–*19*). Compared to niacin, NR with the dose of 1 g/day was less potent in elevating whole-blood NAD^+^ levels as NR induced a twofold increase after 5 months, while niacin with the same dose induced a sixfold increase after 4 months (*5*). However, the levels of NR and niacin were not molar equivalents in these studies, but the molar concentration was higher for niacin. So far, short-term clinical studies have not reported the positive effects of NR on tissue NAD^+^ biosynthesis (*14*–*19*). We here provide the first evidence that long-term NR supplementation increases muscle and WAT NAD^+^ biosynthesis in humans regardless of BMI. This NAD^+^ biosynthesis–boosting effect occurred even in the heavier cotwins from the BMI-discordant pairs, which showed lower expression of NAD^+^ biosynthetic genes, *NMNAT3* and *NRK1*, especially in WAT, compared with the leaner cotwins, at baseline. Given that the up-regulation of both salvage pathway enzymes *NRK1* and *NAMPT* was observed upon NR, especially in muscle, NAD^+^ generated in tissues was likely originating both from NR and NAM, the degradation product of NR. This agrees with mouse studies showing that most of the orally consumed NR is metabolized to NAM, and a small proportion of NR is directly used for NAD^+^ biosynthesis via NRKs (*30*).

Currently, there is plenty of evidence for the efficacy of NR on muscle mitochondrial biogenesis in mice (*7*–*11*) but not in humans (*19*, *22*). In our study, the NR-induced improvement in NAD^+^ metabolism led to an increase in muscle mitochondrial number and mtDNA amount after 5 months. In line with the previous mouse studies (*9*, *31*), the mechanism was linked to the up-regulation of *SIRT1*/*ERR*α/*TFAM/MFN2*. The effect of NR on mitochondrial biogenesis was tissue specific as significant changes in mitochondria-related parameters were not detected in WAT. Collectively, our evidence emphasizes that a supplementation time longer than 3 months is required to detect the impact of NR on muscle mitochondrial biogenesis in healthy individuals with overweight and obesity. This notion is supported by our previous finding showing that niacin with the dose of 1 g/day significantly increased muscle mitochondrial mass in healthy normal-weight controls after 4 months (*5*). Overall, our current clinical trial is a proof of principle of NR’s effects on muscle mitochondrial biogenesis in humans.

NR is known to exert beneficial effects on muscle satellite cells in mice (*10*). Here, we show that NR reduced the number of muscle PAX7^+^ satellite cells likely by increasing differentiation and fusion of satellite cells to the existing myofibers. Given that we did not observe the increase in muscle mass, our data reveal that satellite cell fusion can be uncoupled from muscle hypertrophy under specific conditions in humans. It has been shown previously that during homeostasis, reduced Notch activity in satellite cells results in premature differentiation and fusion to myofibers bypassing self-renewal (*23*). It will be interesting to probe whether NR modulates Notch signaling in satellite cells. Our myoblast results align with a recently published study demonstrating an increased differentiation capacity of NR-supplemented human muscle precursor cells (*32*). One molecular mechanism mediating NR’s effect on myogenic differentiation could be the NR’s degradation product, NAM, which is shown to increase myogenesis in mouse stem cells (*33*). Whether the increased differentiation of satellite cells provides a functional benefit or disadvantage upon exercise and muscle damage needs to be addressed in future studies.

In mice, NR with the dose of 400 to 500 mg/kg per day has been shown to counteract the negative metabolic consequences of a high-fat diet (*9*), but a high dose of NR (~1000 mg/kg per day) has been shown to cause WAT dysfunction and impaired glucose homeostasis (*34*). So far, positive outcomes on body composition from the clinical trials are largely lacking (*14*–*19*). During our study, adiposity increased in the twins from the BMI-discordant pairs, although their self-reported lifestyle factors remained the same. The underlying mechanism may be linked to morphological and molecular changes in WAT. However, we did not detect changes in adipocyte number or size or WAT *PPARγ* dysregulation that have been reported in mice upon high doses of NR (*34*). As the body weight and fat mass tended to increase also in the placebo-treated twins, the weight gain may be related to a normally occurring increase in adiposity over time rather than to NR per se (*35*). In our previous study, the long-term niacin supplementation resulted in an opposite outcome related to body composition compared with the effects of NR in the current study. Niacin with the dose of 1 g/day reduced whole-body fat percentage in normal-weight study participants after 4 months (*5*). Similarly, as for adiposity, no significant effect on insulin sensitivity has been found in the published clinical trials with NR (*16*, *17*, *19*). Here, we report elevated fasting glucose and insulin levels and impaired insulin sensitivity in the oral glucose tolerance test in twins from the BMI-discordant pairs after a 5-month NR supplementation. As niacin is well known to raise fasting glucose levels and impair insulin sensitivity by increasing hepatic gluconeogenesis and by decreasing insulin signaling (*36*), our findings raise the question whether niacin and NR exhibit a similar mechanism of action on glucose metabolism in humans. Overall, on the basis of the current clinical evidence, larger and longer placebo-controlled studies are required to clarify the role of NR as a modifier of adiposity and glucose metabolism in humans. Given that most clinical studies with NR, like ours, have been performed in metabolically healthy individuals (*14*–*16*, *18*, *19*), human studies in patient populations with metabolic diseases are warranted in the future.

Epigenetic mechanisms such as DNA methylation have been shown to regulate gene expression upon NR in mice (*37*). Typically, DNA methylation in gene promoter regions is associated with transcriptional repression. However, the relationship between DNA methylation and gene expression at a given location is not straightforward. Here, we demonstrate that NR supplementation led to a muscle-specific decline in global DNA methylation likely via reduced methyl pool size due to increased elimination of NAM via methylation. Nevertheless, both hyper- and hypomethylation of CpG sites were detected in muscle and WAT, although most of the analyzed sites were hypomethylated upon NR. Notably, methylation changes at the CpG sites were associated with the altered tissue expression for several analyzed genes such as *PGC-1*α, *PAX7*, and *NAMPT*. Given that NAD^+^ precursors have been typically linked to the regulation of gene transcription via NAD^+^-dependent histone deacetylases (*38*), our study underscores that DNA methylation may also be involved in the epigenetic control of tissue metabolism and muscle stem cell identity upon NR in humans. The mechanism via which NR diminishes DNA methylation could be related to the inhibition of DNA methyltransferases through direct or indirect mechanisms, as suggested previously (*37*).

The disruption of the gut microbiota homeostasis, i.e., dysbiosis, is a typical feature of metabolic and muscle diseases (*39*). In line with the previous mouse studies (*11*, *40*), NR ingestion slightly improved the gut microbiota composition in our study participants. Similarly, niacin has been reported to beneficially affect the gut microbiota composition in humans (*41*). We here show that NR supplementation increased the proportion of the only identified species of the genus *Faecalibacterium*, namely, *F. prausnitzii*, which is a gut bacterium that promotes metabolic health and anti-inflammatory responses (*27*, *28*). In agreement with the latter notion, *Faecalibacterium* abundance was negatively associated with high-sensitivity CRP. Intriguingly, our correlation analyses between *Faecalibacterium* abundance and whole-blood NAD metabolites imply that *F. prausnitzii* may compete for the NAD^+^ precursors with the host for its own NAD^+^ biosynthesis and promote the in vivo efficiency of NR in humans. Supporting these conclusions, it is known that *F. prausnitzii* can metabolize NR and NMN (*42*) and that the gut microbiota are crucial for the NAD^+^ boosting effect of NR in mice (*29*). Together, our findings suggest that NR changes the gut microbiota composition in a manner that may improve health and that the NAD^+^ metabolism could be partly regulated through the interaction between the host and *Faecalibacterium* in humans.

In conclusion, NR supplementation is a potential treatment option to be tested in individuals with decreased muscle mitochondrial biogenesis and dysbiosis. As possible adverse effects, we report a declining muscle satellite cell number and a possibility for impaired glucose metabolism. As patients with chronic muscle disease typically exhibit satellite cell dysfunction, muscle performance and regeneration are important endpoints to be monitored in future NR clinical trials. Overall, our data underscore the role of NR as a potent modifier of systemic NAD^+^ levels, muscle mitochondrial biogenesis, satellite cell function, DNA methylation, and the gut microbiota in humans. Notably, NR modulated these metabolic processes similarly in both leaner and heavier cotwins, i.e., regardless of BMI.

### Limitations of the study

The study participants volunteered to two muscle and WAT biopsy collections, yielding material sufficient for histology, electron microscopy, and RNA and DNA isolation, but not for mitochondrial respirometry and protein level analyses. The electron microscopic evaluation of tissues was limited to muscle because of improper fixation of WAT samples. The open study setting and the lack of a statistically powered placebo group may compromise our results related to body composition and metabolic health, and these endpoints need to be followed up in larger placebo-controlled trials. Although NR supplementation significantly increased blood NAD^+^ levels, it is not clear whether the observed effects of NR administration are specific to the boosting of NAD^+^ or other NAD metabolites such as methylNAM. From a mechanistic perspective, we demonstrate that NR supplementation up-regulated *SIRT1*/*ERR*α/*TFAM*/*MFN2* and modulated epigenetic control of gene transcription, but this provides only a correlative insight into the mechanism via which NR regulates mitochondrial biogenesis in humans. Therefore, further work must be done to determine the mechanisms underlying the reported mitochondrial phenotype. In addition, we recognize that it is possible that long-term NR supplementation increases mitochondrial biogenesis as a compensatory mechanism for NR’s toxicity to mitochondria. Consequently, further studies are needed to understand whether long-term NR supplementation is truly beneficial for muscle mitochondrial function, as well as for muscle physiology such as exercise tolerance and regeneration, in humans.

## MATERIALS AND METHODS

### Study design

BMI-discordant and BMI-concordant MZ twin pairs were screened from two large population-based cohorts of Finnish twins (FinnTwin12 and Finntwin16, *n* = 5000, 1200 of which are MZ), established by J.Ka. (*43*) and recruited by K.H.P. This study was approved by the Ethics Committee of the Helsinki University Central Hospital (protocol number 270/13/01/2008), and the study was conducted according to the principles of the Declaration of Helsinki. Written informed consent was obtained from all subjects.

The primary objective of this study was to investigate whether long-term NR supplementation increases muscle and WAT mitochondrial biogenesis in humans. In addition, we aimed to understand whether this intervention could also affect adiposity and metabolic health. Our primary and secondary endpoints were prospectively selected (clinicaltrials.gov entry NCT03951285). A sample size of 10 twin pairs was determined sufficient to detect an effect size of 0.7 with 0.05 significance level and 0.80 power in mitochondrial-related parameters (*44*).

The study participant selection is described in fig. S1. Twenty-two MZ twin pairs with cotwins having a BMI difference of at least 2.5 kg/m^2^ were considered as BMI discordant. Two twin pairs did not meet the inclusion criteria presented in the next paragraph. Another four twin pairs were considered as BMI concordant (within-pair difference in BMI, <2.5 kg/m^2^). Four BMI-discordant twin pairs discontinued the intervention because of pregnancy, muscle pain, or unwillingness to follow the study protocol (fig. S1). Sixteen BMI-discordant and four BMI-concordant twin pairs were included in the data analyses, as shown in fig. S1.

Inclusion criteria were as follows: (i) age > 18 years, (ii) BMI > 18.5 kg/m^2^ in both members of the twin pair, (iii) agreement to maintain current level of physical activity throughout the study, and (iv) agreement to avoid vitamin supplementation or nutritional products with vitamin B3 14 days before the enrolment and during the study. The exclusion criteria were as follows: (i) unstable medical conditions as determined by the investigator, (ii) clinically significant abnormal laboratory results at screening (e.g., aspartate aminotransferase and/or ALT > 2 × upper limit of normal, and/or bilirubin > 2 × upper limit of normal), (iii) subjects who would have had a planned surgery during the course of the trial, (iv) history of or a current diagnosis of any cancer (except for successfully treated basal cell carcinoma diagnosed less than 5 years before screening), (v) history of blood/bleeding disorders, (vi) immunocompromised individuals such as subjects that had undergone organ transplantation or subjects diagnosed with human immunodeficiency virus, (vii) hepatitis, and (viii) blood donation in the previous 2 months.

Data were analyzed from 16 BMI-discordant (44% females) and 4 BMI-concordant twin pairs (50% males) with a mean age of 40 (Table 1). The study participants had the following medications: hypertension (*n* = 2; angiotensin II receptor blocker, calcium channel blocker, and diuretic), asthma or allergy (*n* = 7; an inhaled corticosteroid, beta-2 agonist, and antihistamine), type 2 diabetes (*n* = 1; metformin and insulin), rheumatoid arthritis (*n* = 2; hydroxychloroquine and methotrexate), psychiatric (*n* = 3; selective serotonin intake inhibitor, valproate, a tricyclic antidepressant, and a neuroleptic), and oral contraceptives (*n* = 2). The number of smokers is shown in Table 1.

The examination protocol of this nonrandomized, open-label study is described in Fig. 1A. All twins from the BMI-discordant pairs received NR supplementation. For BMI-concordant twin pairs, one cotwin received NR and the other cotwin received placebo. The cotwins were allocated to the two groups randomly and in a double-blinded fashion. The NR dose (1 g/day) was selected on the basis of a previous study showing that a niacin supplementation of 1 g/day had a favorable effect on systemic NAD^+^ levels, body composition, and mitochondrial biogenesis (*5*). The weekly dose was initially 250 mg/week. The daily NR dose was gradually escalated by 250 mg/week so that the full dose of 1 g/day was reached in 1 month. The supplementation was continued for 4 months with the full dose. At the end of the study, the dose was slowly decreased to the initial level at a rate of 250 mg/week. Study participants were advised to take half of the NR dose in the morning and the other half in the evening. The collection of samples such as blood, WAT and muscle biopsies, saliva, and feces was performed after overnight fasting at the same time in the morning both at baseline and after 5 months. Safety laboratory tests included blood count, iron levels, and liver and kidney function tests. One-to-one phone conversations were conducted at least once a month to follow up compliance to the study and potential symptoms or side effects. All participants were instructed to continue their normal routine with no changes to their physical activity and diet. The analysis of body composition, clinical variables, and whole-blood NAD metabolites was performed for all twin pairs. Gene expression, imaging, and omic analyses were conducted only for BMI-discordant twin pairs.

### Body composition, energy intake, and expenditure

Weight, height, waist circumference, whole-body fat (assessed by dual-energy x-ray absorptiometric scans), abdominal subcutaneous and visceral fat (by magnetic resonance imaging), and liver fat (by magnetic resonance spectroscopy) were measured in all study participants as described previously (*45*). In short, imaging for amounts of subcutaneous adipose tissue, visceral adipose tissue, and liver fat was performed on a clinical 1.5-T imager (Avanto, Siemens, Erlangen, Germany). To allow measurement of abdominal fat distribution, a stack of abdominal T1-weighted magnetic resonance images (16 slices, slice thickness of 10 mm, repetition time of 91 ms, time to echo of 5.2 ms, and flip angle of 80°) were obtained from 8 cm above to 8 cm below the L4/5 lumbar intervertebral disks using frequency selective fat excitation. The amount of visceral and subcutaneous adipose tissue was determined using the segmentation software SliceOmatic 5.0 (TomoVision, Quebec, Canada).

A point-resolved spectroscopy sequence was used for volume selection in hepatic magnetic resonance spectroscopy. A 25 mm × 25 mm × 25 mm voxel was placed in the middle of the right liver lobe, and liver spectra with time to echo of 30 ms and four averages were collected. Signal acquisition was triggered to end exhalation using a navigator belt to eliminate artifacts due to respiratory motion so that repetition time was kept at >4000 ms. Liver spectra were analyzed with jMRUI 6.0 software (*46*), and intensities of methylene and water resonances were determined using the AMARES algorithm (*47*). Signal intensities were corrected for relaxation effects, and liver fat was determined as an intensity ratio of methylene/(methylene + water). Ratios were further converted to mass fractions as described previously (*48*).

Muscle mass and energy expenditure were measured using the Tanita MC-980 bioelectrical impedance device. Physical activity was estimated with the Baecke questionnaire, and dietary intake from 3-day food records was analyzed with the Diet32 program (Aivo Finland Oy) based on a national Finnish database for food composition (Fineli; www.fineli.fi).

### Blood laboratory examinations

Routine blood tests were performed for all study participants. Blood samples were collected after overnight fasting, and whole-blood, separated plasma, and serum samples were frozen at −80°C. Blood count, ALT, aspartate transaminase, hemoglobin A1c, glucose, insulin, C-peptide, lactate, total cholesterol, high-density lipoprotein, low-density lipoprotein, triglycerides, homocysteine, and high-sensitivity CRP were analyzed using standardized methods at the HUSLAB laboratories.

A 75-g oral glucose tolerance test with four time points (0, 30, 60, and 120 min) was performed after a 12-hour overnight fast, followed by measurements of plasma glucose with spectrophotometric hexokinase and glucose-6-phosphate dehydrogenase assay (Roche Diagnostics) and of serum insulin with time-resolved immunofluorometric assay (PerkinElmer). Free fatty acids were determined with a NEFA kit (Wako Chemicals #999-75406), apolipoprotein B levels with an Apolipoprotein B Konelab kit (Thermo Fisher Scientific, #981663), and plasma adiponectin levels with an enzyme-linked immunosorbent assay (ELISA) kit (R&D Systems, #DHWAD0).

### Adipose tissue sampling

Adipose tissue biopsies were collected from all study participants at baseline and at the end of the study (Fig. 1A). The subcutaneous WAT biopsies were taken from superficial abdominal adipose tissue using a needle biopsy under local anesthesia (lidocaine). WAT samples for analyses were snap-frozen and stored in liquid nitrogen or at −80°C.

Samples used for determination of adipocyte size and number were immediately processed by collagen digestion and separation of adipocytes by centrifugation as described previously (*49*). In brief, the subcutaneous adipose tissue was minced and incubated for 1 hour at 37°C with continuous shaking in 10 ml of adipocyte medium [Dulbecco’s modified Eagle’s medium/F-12 (1:1; Invitrogen) with 16 μM biotin, 18 μM panthotenate, 100 μM ascorbate, and antibiotic-antimycotic (Invitrogen)], supplemented with 2% bovine serum albumin (Sigma-Aldrich) and collagenase A (2 mg/ml; Roche Diagnostics). Digestion was terminated by adding adipocyte medium supplemented with 10% newborn calf serum (Sigma-Aldrich) and centrifuging for 10 min at 600*g*. After washing the adipocytes with adipocyte medium, images of the adipocytes were taken with a light microscope (Zeiss, Axioplan2) using ×50 magnification. Adipocyte diameters were automatically measured with a custom algorithm for ImageJ (*50*), which preprocessed the image to enhance the adipocyte borders and used a circle-detection algorithm to identify the cells. The algorithm identified the adipocytes with standardized microscope settings and was validated against 2000 manually measured diameters from 20 pictures (*r* = 0.85, *P* < 0.001). Mean adipocyte volume was calculated for each individual using the following formula

$$V=\frac{\sum_{1}^{100}\left(\frac{\pi \;\cdot \;d_{i}^{3}}{6}\right)}{100},w=\frac{\sum_{1}^{100}\left(\frac{\pi \;\cdot \;d_{i}^{3}\;\cdot \;0.915}{6\;\cdot \;10\hat{6}}\right)}{100},n=\frac{m\;(\mathrm{kg})}{V\;(dm3)\cdot 0.915\;\left(\frac{\mathrm{kg}}{dm3}\right)}$$

*V* = cell volume (μm^3^), *d* = cell diameter (μm), *w* = cell weight (μg), and *n* = total adipocyte number, where *m* = total body fat mass (kg) and *V* (m^3^) ∗ rho (density, fat) = mean weight of a single adipocyte. Density of fat cell triglycerides = 0.915 g/ml. Adipocytes were assumed to be spheres.

### Muscle sampling, TEM, and satellite cell histology and quantification

Muscle tissue biopsies were collected from all study participants at baseline and at the end of the study (Fig. 1A) via Bergström needle biopsy from the vastus lateralis muscle in sterile conditions under local anesthesia. The muscle samples for molecular analyses were snap-frozen and stored in liquid nitrogen or at −80°C.

Samples for TEM analysis were fixed in 2.5% glutaraldehyde. For plastic embedding, they were then treated with 1% osmium tetroxide dehydrated in ethanol and embedded in epoxy resin. One-millimeter section was stained with methylene blue (0.5%, w/v) and boric acid (1%, w/v) and examined with a light microscope to mark the interesting areas for TEM analyses. Thereafter, 60- to 90-nm sections were cut on grids and stained with uranyl acetate and lead citrate by the Viikki Electron Microscopy Unit of the Institute of Biotechnology (EMBI, Biocenter Finland) and viewed with a JEM-1400 transmission electron microscope (Jeol). From each study subject’s pre- and post-NR time points, 15 TEM images were taken from the intermyofibrillar area of type I muscle fibers, determined by mitochondria content, using ×2000 magnification. Total mitochondria number per 10 μm^2^ relative to the muscle fiber area, mitochondrial surface area per muscle fiber area (%), dimension values [average diameter (μm) and perimeter (μm)], and total muscle fiber area (μm^2^) were determined by painting each mitochondrion in Microscopy Image Browser (*51*). These values were used to calculate the total mitochondria number per 10 μm^2^ of muscle fiber area, mitochondrial surface area per muscle fiber area (%, mitochondria density), and major shape descriptors, branching (form factor) and length-to-width ratio (aspect ratio).

Muscle samples for histological staining were snap-frozen in liquid nitrogen–cooled isopentane. Cryosections were prepared to measure PAX7 immunostaining in muscle tissues: Nine-micrometer-thick frozen sections were postfixed in 4% paraformaldehyde in Dulbecco’s phosphate-buffered saline (DPBS) for 10 min at room temperature and washed three times for 5 min in DPBS. Slides were then incubated overnight at 4°C in mouse monoclonal Pax7 primary antibody (Developmental Studies Hybridoma Bank, #AB_528428, deposited by Kawakami A) diluted 1:100 in blocking solution (15% normal goat serum, 1.5% bovine serum albumin, and 0.5% Triton X-100 in DPBS). Slides were washed three times for 10 min in DPBS and incubated in Cy3 goat anti-mouse immunoglobulin G1 secondary antibody (1:500 dilution; Jackson ImmunoResearch, #115-165-003) and Hoechst (1 μg/ml) for 1 hour at room temperature to visualize the nuclei. Slides were washed as described above and mounted. Images were generated using Pannoramic 250 FLASH II digital slide scanner (3DHistec, Thermo Fisher Scientific) and Leica SP8 STED confocal microscope (Leica). For PAX7^+^ cell quantification, muscle sections were scored from a minimum of two to three sections per sample per condition and normalized to cross-sectional area of the muscle section. All quantifications were performed using Case Viewer 2.0 software (3DHistech, Thermo Fisher Scientific), and images were blinded for quantification.

### Myoblast cell cultures

Muscle samples for primary muscle cell cultures, i.e., myoblast cultures, were processed immediately after excision. Samples were washed with 5 ml of isolation medium [0.05% trypsin-EDTA (SAFC Biosciences), glucose (1.8 g/liter; Sigma-Aldrich), *N*-2-hydroxyethylpiperazine-*N*-2-ethane sulfonic acid buffer (7.15 g/liter; Sigma-Aldrich), NaCl (7.6 g/liter; Sigma-Aldrich), KCl (0.224 g/liter; Sigma-Aldrich), and phenol red (1.2 mg/liter; Sigma-Aldrich)]. Thereafter, 5 ml of isolation medium was added and tissue was minced. Another 10 ml of isolation medium was added, and the sample was transferred to a 20-ml beaker with a magnetic stirrer and placed in a 37°C incubator for 20 min. The supernatant containing muscle cells was transferred into a 50-ml centrifuge tube containing 15 ml of myoblast growth medium [Ham’s F10 with GlutaMAX (Gibco) supplemented with 15% fetal bovine serum (Thermo Fisher Scientific), fetuin (500 mg/liter; Sigma-Aldrich), insulin (200 mg/liter; Sigma-Aldrich), 0.05% bovine serum albumin (Sigma-Aldrich), dexamethasone (0.39 mg/liter; Sigma-Aldrich), gentamicin (50 mg/liter; Sigma-Aldrich), and human epidermal growth factor (2 mg/liter; Thermo Fisher Scientific)]. This process was repeated twice to break down any remaining tissue. Last, the supernatant containing the isolated muscle cells was centrifuged for 10 min at 100*g* to collect the cells, and cell pellet was resuspended in 5 ml of myoblast growth medium. Myoblasts were seeded on 25-cm^2^ flasks (Corning). After reaching 70% confluence, they were subcultured in 150-cm^2^ flasks (Corning) and finally on six-well plates (Corning) at a density of 100,000 cells per well. When confluent, the cells were collected and stored at −80°C.

### Targeted quantitative NAD metabolome analysis

The measurement of whole-blood NAD metabolome was carried out from all study participants after dual extractions as recently described (*5*). For analysis of Me4py (group A analyte), samples were spiked with 400 pmol of [d3]-Me4py (internal standard A). For analysis of NAD^+^, NADP, NMN, NAR, NAAD, and ADPR, samples were dosed with 13C-yeast extract (internal standard B) as described (*18*, *52*).

In short, to quantify Me4py (group A analyte), 75 μl of whole blood was added to 20 μl of group A internal standard and 500 μl of 3:1 4% trichloroacetic acid:acetonitrile was added. The mixture was allowed to sit on ice for 20 min, after which samples were sonicated twice for 20 s and centrifuged at 4°C for 13 min at 16,100*g*. Next, the supernatant was removed and dried under vacuum overnight at room temperature. The samples were reconstituted in 2% acetonitrile/water immediately before the analytical run. To quantify NAD^+^, NADP, NMN, NAR, NAAD, and ADPR (group B analytes), 75 μl of whole blood was added to 20 μl of group B internal standard prepared in water and mixed with 500 μl of 3:1 4% trichloroacetic acid:acetonitrile with vortexing. After resting on ice, the samples were centrifuged as described above. Next, supernatant was removed and dried under vacuum overnight and reconstituted in 2% acetonitrile/water. After reconstitution, samples were transferred to Waters polypropylene plastic total recovery vials and stored in a Waters Acquity H class autosampler maintained at 8°C until injection. In all cases, 8 μl of extract was loaded onto the column. For one set of samples, group B analytes plus Me4py were extracted as for the group B analytes. One hundred microliters of blood was used, and 200 pmol of d3-Me4py was added to the internal standard mix. All analytes in this group except NAAD had a corresponding stable labeled internal standard. Labeled NAD^+^ was used as the internal standard for NAAD.

Separation and quantitation of analytes were performed with a Waters Acquity LC interfaced with a Waters TQD mass spectrometer operated in positive ion multiple reaction monitoring mode as described (*52*) with minor modifications. Multiple reaction monitoring transitions monitored were as follows: NAD^+^ 664.1>136.1; 13C10-NAD^+^ 674.1>136.1; NADP 744.1>136.1; 13C10-NADP 754.1>136.1; NAAD 665.1>136.1; NMN 335>123: 13C5-NMN 340>123; NAR 256>124; 13C5-NAR 261>124; ADPR 560>136.1; 13C10-ADPR 570>136.1; Me-4-py 153>136; d3-Me-4-py 156>139. Separate Hypercarb columns (Thermo Fisher Scientific) were used for the separations. The conditions for group A were as follows: solvent A: 10 mM NH_4_OAc with 0.1% formic acid, solvent B: acetonitrile with 0.1% formic acid, and solvent D: methanol, flow of 0.30 ml/min; gradient initial of 98% A and 2% B; 2.25 min, 98% A and 2% B; 11 min, 74.8% A, 17.9% B, and 7.3% D; 11.1 min,10% A and 90% B; 14.3 min, 10% A and 90% B; 14.4 min, 98.2% A and 2% B; end, 18.5 min. Conditions for group B were as follows: solvent A: 7.5 mM NH_4_OAc with 0.05% NH_4_OH and solvent B: acetonitrile with 0.05% NH_4_OH; flow of 0.353 ml/min; gradient initial of 97% A and 3% B; 1.8 min, 97% A and 3% B; 10 min, 65.5% A and 34.5% B; 11 min, 10% A and 90% B; 13.2 min, 10% A and 90% B; 13.3 min, 97% A and 3% B; end, 19 min.

### MtDNA analyses and quantification

Total DNA, including mtDNA, was extracted from the snap-frozen muscle (15 mg) and WAT (150 mg) using standard phenol-chloroform extraction and ethanol precipitation and stored at 4°C until use. MtDNA content was determined by the ratio of mtDNA to genomic DNA using RT-qPCR. To detect genomic DNA, primers for nuclear β-amyloid precursor protein, beta-2-microglobulin, and hemoglobin subunit beta were used. To measure mtDNA, mitochondrial 16*S* rRNA, cytochrome B, and D-loop region primers were chosen. The PCR primer sequences are provided in table S10.

RT-qPCR was performed using SYBR Green MasterMix (Thermo Fisher Scientific), 10 μM primers, and 2 ng of DNA template in a 384-well plate format using CFX384 Touch Real-Time PCR Detection Systems (Bio-Rad Laboratories). Thermal cycling included initial denaturation of 3 min at 95°C, 39 cycles of 10 s at 95°C and 30 s at 62°C, final extension of 10 s at 95°C, and melting curve analysis from 65° to 95°C with 0.5°C increments. MtDNA content was presented as a relative level of mtDNA genome per nuclear genome. Data analysis was performed in qBASE+ 3.2 software (Biogazelle) using the ΔΔCt method.

### Gene expression

Total RNA from myoblasts was isolated using TRIzol reagent (Invitrogen) according to the manufacturer’s instructions. Total RNA from muscle tissue and WAT was isolated using the AllPrep DNA/RNA/miRNA Universal Kit (Qiagen) according to the manufacturer’s instructions with minor modifications. In short, muscle tissue (30 mg in 600 μl of Buffer RLT Plus) was lysed using TissueLyser II (Qiagen) at 25 Hz for 2 min, and the lysate was transferred into the AllPrep DNA Mini spin column. WAT (250 mg in 1 ml of Buffer RLT Plus) was similarly lysed except for the time being 2 × 2 min. As the sample volume exceeded the recommended amount, the lysate was divided into two AllPrep DNA Mini spin columns. Thereafter, muscle and WAT samples were processed according to the manufacturer’s instructions. RNA was stored at −80°C.

Complementary DNA (cDNA) was synthesized from 0.5 μg of muscle tissue and myoblast RNA using the QuantiTect Reverse Transcription Kit (Qiagen) according to the manufacturer’s instructions. The SuperScript VILO cDNA Synthesis Kit (Thermo Fisher Scientific) was used to synthesize cDNA from 0.5 μg of WAT RNA according to the manufacturer’s instructions.

Expression levels of genes associated with NAD^+^ biosynthesis, mitochondrial metabolism, mitochondrial OXPHOS, and additional potential NR target genes were determined from tissue samples with RT-qPCR. Gene expression levels were measured from myoblasts to assess their self-renewal, differentiation, and fusion potential. The RT-qPCR primer sequences are provided in table S10. RT-qPCR was performed using SYBR Green MasterMix (Thermo Fisher Scientific), 10 μM primers, and 5 ng of DNA template in 384-well plate format with conditions identical to those used for mtDNA quantification PCR. Three reference genes were used for normalization: actin, beta-2-microglobulin, and tyrosine 3-monooxygenase/tryptophan 5-monooxygenase activation protein zeta. Data analysis was performed with the ΔΔCt method using the qBASE+ 3.2 software (Biogazelle).

### DNA methylation analysis

High–molecular weight WAT and muscle DNA was extracted using a QIAmp DNA Mini kit (Qiagen) and bisulfite-converted by an EZ DNA Methylation kit (ZYMO Research) following the manufacturers’ protocols. DNA methylation was quantified using Infinium MethylationEPIC BeadChip arrays (Illumina) according to the manufacturer’s instructions. The data were preprocessed using R-package minfi (*53*). Sample quality control was performed in R-package MethylAid with default settings (*54*). Bad-quality probes were removed with the following criteria: (i) detection *P* > 0.01, (ii) bead counts of <3, (iii) zero methylation probe intensity, and (iv) ambiguously mapping probes (*55*). The data were normalized using functional normalization (*56*) followed by Beta Mixture Quantile normalization (*57*). CpG sites annotated to genes with gene expression data available were selected for the analysis, resulting in a final dataset of 518 CpGs in WAT and 619 in muscle.

The average long interspersed nuclear element-1 methylation levels were computed using R-package REMP (*58*) for each individual as a surrogate measure for global DNA methylation level. Differences in global methylation levels upon NR (baseline versus 5 months) in the twins from the BMI-discordant pairs and differences in the changes of global methylation upon NR between the leaner and the heavier cotwins were compared using Wilcoxon signed-rank test. The level of significance (two-tailed) was set at *P* < 0.05.

Differential methylation analysis was performed for the selected CpGs in WAT (*n* = 14 twin pairs/28 individuals) and muscle (*n* = 12 twin pairs/25 individuals) using a linear model (package limma in R-Bioconductor) to identify CpG sites with altered methylation (Benjamini-Hochberg FDR *P* < 0.05) upon NR supplementation in the twins from the BMI-discordant pairs. The regression models were adjusted for, sex, age, smoking, and twinship. Regression analysis was also performed to identify within-pair differences in the changes in CpG site methylation levels (changes from baseline to 5 months, FDR *P* < 0.05).

### Plasma metabolite isolation and nontargeted metabolite profiling

We performed nontargeted metabolite profiling using two different chromatographic–mass spectrometric techniques coupled online to high-resolution mass spectrometry and further acquired data with positive and negative electrospray ionization (ESI) mode. Further information about the instruments’ setup and data acquisition parameters, in concert with molecular feature finding and peek picking, can be obtained from the previous publications (*59*, *60*).

Briefly, plasma samples were thawed on ice, and a 100-μl aliquot of plasma was dispensed into a 96-well Captiva ND filter plate 0.2 μm PP (Captiva ND, 0.2 μm PP, Agilent Technologies) containing 400 μl of ice-cold acetonitrile. Samples were mixed with a pipette to thoroughly precipitate plasma proteins. Samples were then centrifuged at 700*g* for 5 min at 4°C, and the supernatants were collected to a 96-well storage plate and stored at −10°C.

A pooled sample from all biological samples per experiment was injected at the beginning of the sequence to equilibrate the analytical platform and then after every 12 samples throughout the analysis for quality control. In addition, a solvent blank was prepared and injected at the beginning of the sequence. Samples were randomized before the analysis.

The analysis of amphiphilic metabolites was carried out using an ultrahigh-performance liquid chromatography (Vanquish Flex UHPLC system, Thermo Fisher Scientific, Bremen, Germany) coupled online to high-resolution mass spectrometry (Q Exactive Classic, Thermo Fisher Scientific, Bremen, Germany) (*59*). The sample solution (2 μl) was injected onto a reversed-phase column (Zorbax Eclipse XDBC18, 2.1 × 100 mm, 1.8 μm; Agilent Technologies, Palo Alto, CA, USA) that was kept at 40°C. The mobile phase, delivered at 400 μl/min, consisted of water (eluent A) and methanol (eluent B), both containing 0.1% (v/v) of formic acid. The following gradient profile was used: 0 to 10 min: 2 to 100% B, 10 to 14.50 min: 100% B, 14.50 to 14.51 min: 100 to 2% B, and 14.51 to 20 min: 2% B. The sample tray was at 10°C during these analyses. Mass spectrometry was equipped with heated ESI. The positive and negative ionization modes were used to acquire the data. At the beginning and end of the sample analysis, data-dependent product ion scans (MS2) were acquired for each mode. The detector was calibrated before the sample sequence and subsequently operated at high mass accuracy [<2 parts per million (ppm)]. Continuous mass axis calibration was performed by monitoring reference ions *m*/*z* (mass/charge ratio) 214.08963 in the positive ionization mode.

For the analysis of hydrophilic compounds, an ultrahigh-performance liquid chromatography (1290 LC system, Agilent Technologies, Waldbronn, Karlsruhe, Germany) coupled online to high-resolution mass spectrometry (6540 UHD accurate-mass quadrupole-time-of-flight mass spectrometry, Agilent Technologies, Waldbronn, Karlsruhe, Germany) was used (*60*). The sample solution (2 μl) was injected onto a column (HILIC, Acquity UPLC BEH Amide 1.7 μm, 2.1 × 100 mm; Waters Corporation, Milford, MA, USA) that was kept at 45°C. Mobile phases, delivered at 600 μl/min, consisted of 50% (v/v) (eluent A) and 90% (v/v) (eluent B) acetonitrile, respectively, both containing 20 mM ammonium formate (pH 3). The following gradient profile was used: 0 to 2.5 min: 100% B, 2.5 to 10 min: 100% B → 0% B, 10 to 10.1 min: 0% B → 100% B, and 10.1 to 12.5 min: 100% B. The sample tray was at 10°C during these analyses. Mass spectrometry was equipped with a heated ESI source, operated in both positive and negative ionization modes. For data acquisition, a 2-GHz extended dynamic range mode was used in both positive and negative ion modes from 50 to 1600 (*m*/*z*). At the beginning and end of the sample analysis, MS2 values were acquired for each mode. The detector was calibrated before the sample sequence and subsequently operated at high accuracy (<2 ppm). Continuous mass axis calibration was performed by monitoring two reference ions from an infusion solution throughout the runs. The reference ions were *m*/*z* 121.050873 and *m*/*z* 922.009798 in the positive mode and *m*/*z* 112.985587 and *m*/*z* 966.000725 in the negative mode.

### Differential plasma metabolite profile analysis and metabolite identification

Differential metabolite analysis was performed on the plasma metabolite levels of the twins from the BMI-discordant pairs (*n* = 14 twin pairs/28 individuals) using a linear model (package limma in R-Bioconductor) to identify metabolite features that had significantly changed levels (nominal *P* < 0.05) upon NR supplementation in the twins from the BMI-discordant pairs (regardless of heavy/lean status). Regression models were adjusted for leaner/heavier status, sex, age, smoking status, and twinship (shared genetic effects). Regression analysis was also carried out to identify changes in metabolite levels (changes from baseline to 5 months, nominal *P* < 0.05) that were different in the leaner and the heavier cotwins. The regression models were adjusted for sex, age, smoking status, and twinship.

The final dataset contained 580 and 2398 molecular features from hydrophilic interaction chromatography and reversed-phase analysis, respectively. Annotation of these significantly different metabolites found between the study groups was generated on the basis of accurate mass and isotope information, i.e., ratios, abundances, and spacing, as well as product ion spectra (MS2) against existing libraries, either in-house for level I or online spectral databases (i.e., Human Metabolome Database, MzCloud, METLIN, and ChemSpider) for levels II to IV, according to the guidelines from the Metabolomics Standard Initiative (*61*).

### Gut microbiota sequencing and composition analyses

For stool collection at home, study subjects were provided with the supplies and instructions for the collection. Study subjects were asked to keep the samples in a cold environment and to bring the sample to the hospital. The stool was then immediately stored at −80°C. The interval between collecting and freezing the stool was within 12 to 24 hours. The sample collection at the hospital was conducted in the same fashion. For the analyses of the gut microbiota, the exclusion criteria were antibiotic course 1 month before sampling.

For the microbial community analysis, the total microbial DNA was extracted from ~80 mg of feces using Stool Extraction Kit v2 (Hain Lifescience GmbH, Nehren, Germany) and semiautomated GenoXtract (Hain Lifescience Gmbh), accompanied with preceding bead beating. Thereafter, the rRNA gene was amplified using universal primers M13_S-D-Bact-0341-b-S-17 (5′-TGTAAAACGACGGCCAGT-3′) and P1_S-D-Bact-0785-a-A-21 (5′-CCTCTCTATGGGCAGTCGGTGAT-3′) targeting the V3-V4 regions of the small subunit (SSU) rRNA gene. PCR and adding of the linker and fusion primers [0.05 μM M13_S-D-Bact-0341-b-S-17, 0.5 μM IonA_IonXpressBarcode_M13 (Thermo Fisher Scientific, #4474517), and 0.5 μM P1_S-D-Bact-0785-a-A-21] were performed as described previously (*62*). After purifying the PCR products with AMPure XP (Beckman Coulter) and pooling them in equimolar quantities, sequencing was performed as described before (*62*). The 16*S* rRNA gene sequences were quality-filtered and clustered to OTUs at 97% similarity using the CLC Microbial Genomics Package (Qiagen).

The rRNA gene sequences were classified using the SILVA SSU Ref database (99%, arb-silva.de/projects/ssu-ref-nr/). Statistical analyses for the gut microbiota were performed with the CLC Microbial Genomics Package and SPSS Statistics (IBM). The alpha-diversity of the gut microbiota was quantified with the Shannon index, and beta-diversity analysis was based on Bray-Curtis distance and permutational multivariate analysis of variance (PERMANOVA) for significance testing. The taxonomic differences between baseline and 5 months were analyzed with paired Wilcoxon signed-rank test in SPSS, followed by Benjamini-Hochberg FDR correction for multiple testing. The level of significance (two-tailed) was set at *P* < 0.05 after the multiple testing correction.

Microbiota data were further preprocessed and normalized using packages phyloseq and edgeR in R-Bioconductor. Differential analysis was performed for the gut microbiota levels of the BMI-discordant twins (*n* = 11 twin pairs/22 individuals) using a linear model (package limma in R-Bioconductor) (*63*) to identify microbial taxa that significantly changed (FDR *P* < 0.05) upon NR (baseline versus 5 months) in the twins from the BMI-discordant pairs (regardless of heavy/lean status). The regression models were adjusted for leaner/heavier status, sex, age, smoking status, and twinship. Regression analysis was also carried out to identify changes in microbiota (changes from baseline to 5 months, FDR *P* < 0.05) that were different in the leaner and the heavier cotwins as a result of NR intervention. The regression models were adjusted for sex, age, smoking status, and twinship.

*F. prausnitzii* RT-qPCR analysis was performed using iQ SYBR Supermix and CFX96 Real-time PCR Detection System (Bio-Rad Laboratories, Hercules, CA, USA) as described previously (*62*). The RT-qPCR program for the 16*S* rRNA genomic region was preincubation at 95°C for 3 min, 40 cycles at 95°C for 30 s, 53°C for 30 s, and 72°C for 30 s. The results of *F. prausnitzii* were normalized to the results of 16*S* rRNA, and the fold change at baseline and 5 months was calculated with the ΔΔCt method. Because of great interindividual variation, the changes in the abundance of *F. prausnitzii* upon NR (baseline versus 5 months) were analyzed with Cohen’s *d* as follows

$$d=\frac{M_{1}-M_{2}}{{\mathrm{SD}}_{\mathrm{pooled}}}\;{\mathrm{SD}}_{\mathrm{pooled}}=\sqrt{\frac{{\mathrm{SD}}_{1}^{2}+{\mathrm{SD}}_{2}^{2}}{2}}$$

where *M*_1_ and *M*_2_ are the means for the first and second samples, and SD_pooled_ is the pooled SD for the samples.

### Association analyses

Associations between CpG methylation (outcome variable) and gene expression (explanatory variables) were identified. First, using linear models with adjustments for age, sex, smoking status, and twinship, gene expression changes between baseline and 5 months were regressed against DNA methylation changes between baseline and 5 months. Benjamini-Hochberg FDR–corrected *P* < 0.05 was considered significant.

Next, the associations between NAD/plasma metabolites (outcome variable) and the gut microbiota (explanatory variables) were examined. First, changes between baseline and 5 months in NAD metabolites corresponding to changes between baseline and 5 months in the gut microbiota were identified. Then, we identified NAD metabolite changes between baseline and 5 months associated to the baseline gut microbiota. Last, changes between baseline and 5 months in plasma metabolites and in blood clinical parameters, in relation to changes between baseline and 5 months in the gut microbiota, were identified. All the regression models mentioned above were adjusted for leaner/heavier status, sex, age, antibiotics usage, smoking status, and twinship.

### Statistical analysis

Clinical variables, blood NAD metabolites, mitochondrial measures, and gene expression data are expressed as means ± SD for normally distributed variables and as median (interquartile range) for non-normal variables. The normality of the distribution was determined using the Shapiro-Wilk test. Variables with a *P* value of less than 0.05 were considered non-normally distributed. Outlier removal for gene expression and mtDNA amount data was performed in GraphPad Prism by using outlier analysis ROUT (*Q* = 1). Baseline differences between the cotwins (the leaner versus the heavier cotwins or the NR- versus placebo-treated cotwins), changes upon NR or placebo in all individuals (baseline versus 5 months), and differences in the cotwin responses to NR (BMI-discordant pairs) were assessed using paired Wilcoxon signed-rank test using R software, and the level of significance (two-tailed) was set at *P* < 0.05.
