# Supplementary material for: Nicotinamide riboside improves muscle mitochondrial biogenesis, satellite cell differentiation, and gut microbiota in a twin study
Source: Sci Adv. 2023 Jan 13;9(2):eadd5163. doi: 10.1126/sciadv.add5163 (PMC9839336; doi:10.1126/sciadv.add5163)
Supplement: Supplementary file 1 — Figs. S1 to S3 Tables S1, S8 to S10 [file sciadv.add5163_sm.pdf]

Science Advances
AAAS

Supplementary Materials for

Nicotinamide riboside improves muscle mitochondrial biogenesis, satellite cell differentiation, and gut microbiota in a twin study

Helena A. K. Lapatto *et al.*

Corresponding author: Eija Pirinen, eija.pirinen@helsinki.fi; Kirsi H. Pietiläinen, kirsi.pietilainen@helsinki.fi

Sci. Adv. **9**, eadd5163 (2022)
DOI: 10.1126/sciadv.add5163

The PDF file includes:

Figs. S1 to S3
Tables S1, S8 to S10

Other Supplementary Material for this manuscript includes the following:

Tables S2 to S7

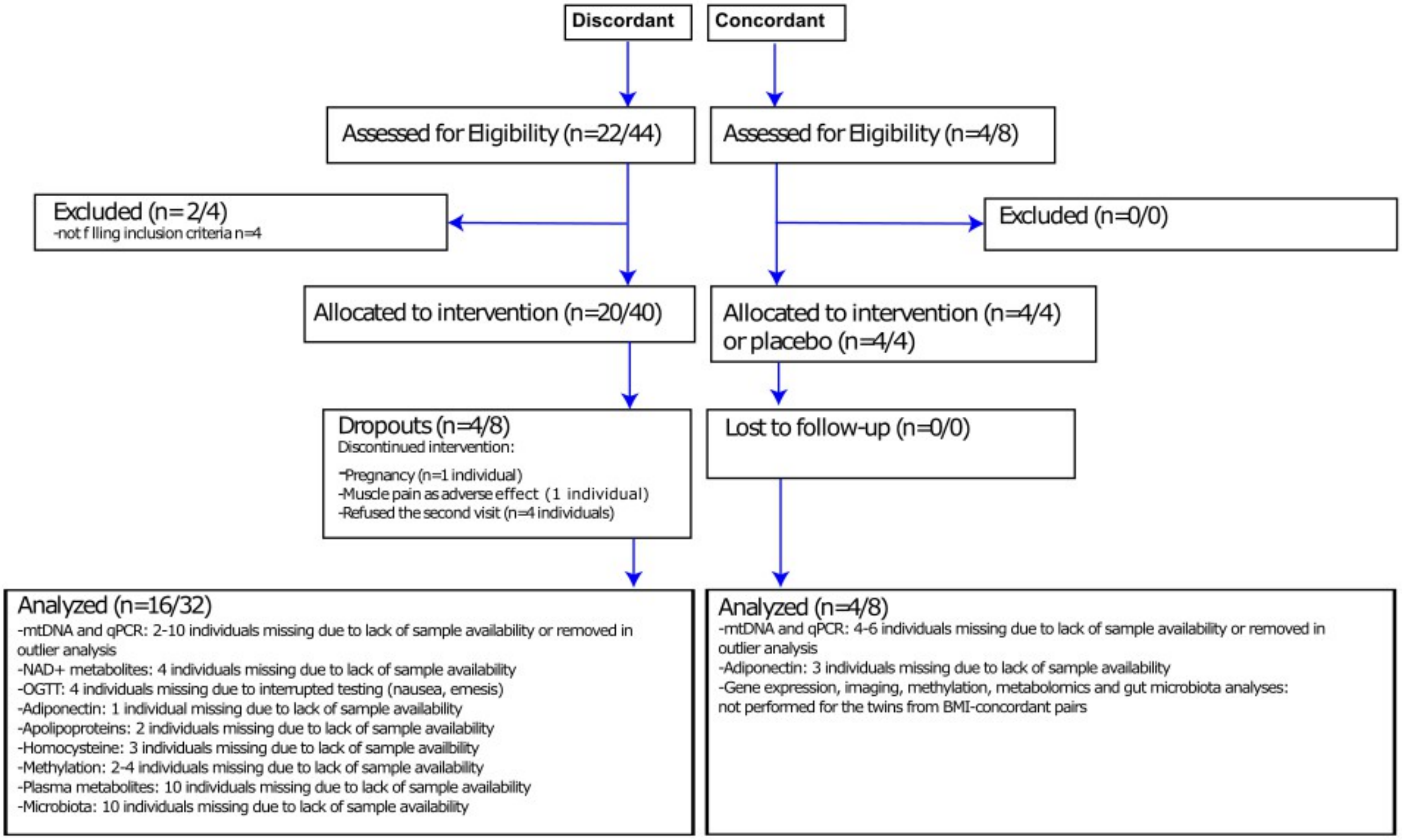

Fig. S1. Flow chart illustrating the procedures for the selection of study participants and data analyses. Related to Fig.1A. Presented as (n=number of twin pairs/number of individuals).

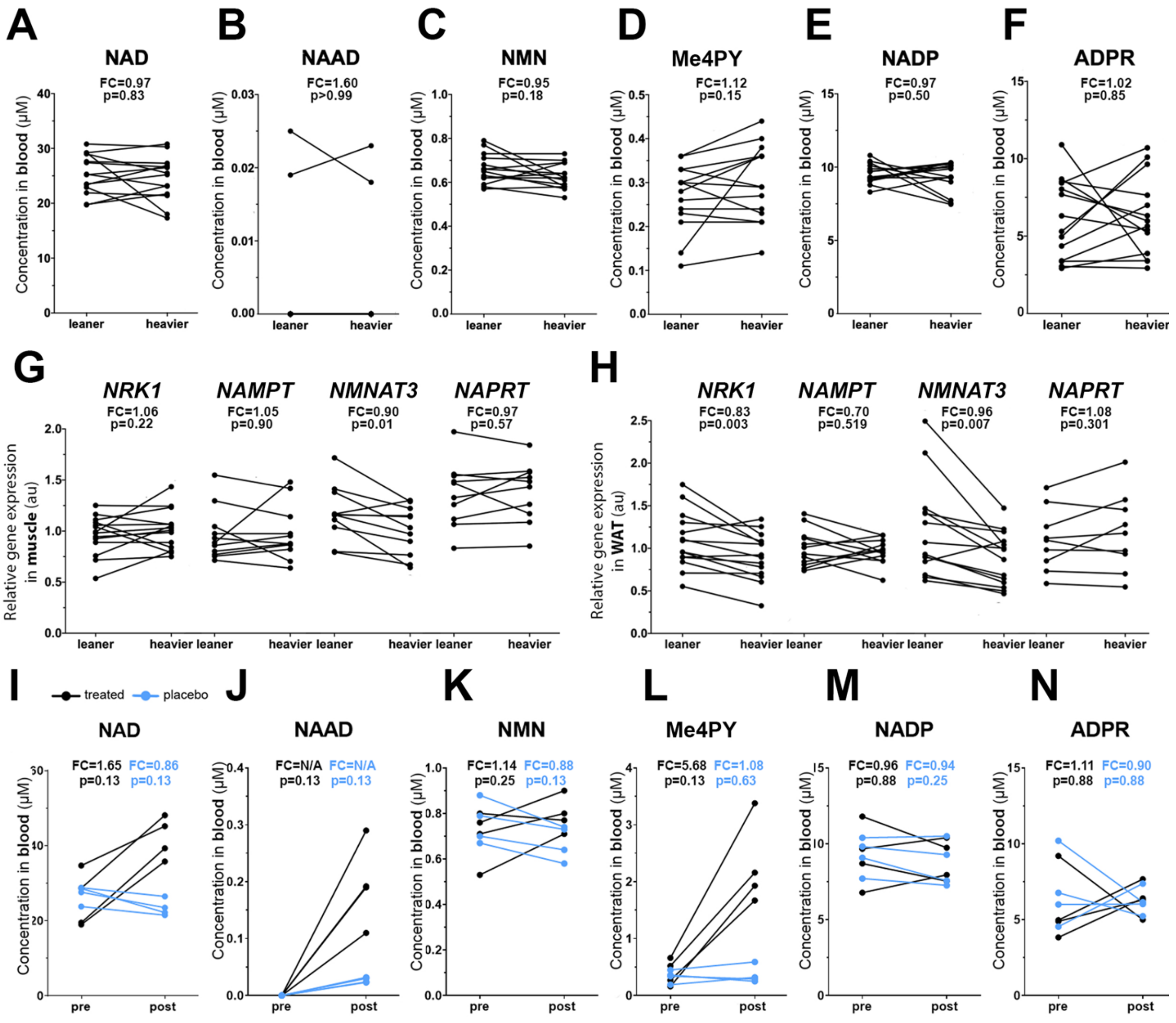

Fig. S2. Baseline NAD$^+$ metabolism in the twins from the BMI-discordant pairs and the effect of placebo and NR treatments on NAD metabolome in the twins from the BMI-concordant pairs. Related to Fig. 1. Number of analyzed study participants is presented as n=number of full twin pairs/number of individuals.

(A-F) The concentration of whole blood NAD metabolites at baseline in the twins from the BMI-discordant pairs (n=14 pairs/28 individuals).

(G) Expression of genes associated with NAD$^+$ biosynthesis in muscle at baseline in the twins from the BMI-discordant pairs (n=11-15 pairs/22-30 individuals).

(H) Expression of genes associated with NAD$^+$ biosynthesis in WAT at baseline in the twins from the BMI-discordant pairs (n= 9-14 pairs/18-28 individuals).

(I-N) The concentration of whole blood NAD metabolites in placebo and NR-treated BMI-concordant twin pairs (n=4 pairs/8 individuals). One cotwin of the twin pair was treated with NR (in black) and the other cotwin with placebo (in blue).

Pre and post value of each individual is connected with a line. Fold change (FC) indicates either the mean of the heavier cotwin values divided with the leaner cotwin values (baseline graphs) or the post NR values divided by the pre NR values (pre versus post graphs). FC could not be

calculated for NAAD as its concentration at baseline was zero. Arbitrary unit (au) indicates relative gene expression normalized to the expression of reference genes. Paired Wilcoxon signed-rank test was used as the statistical test.

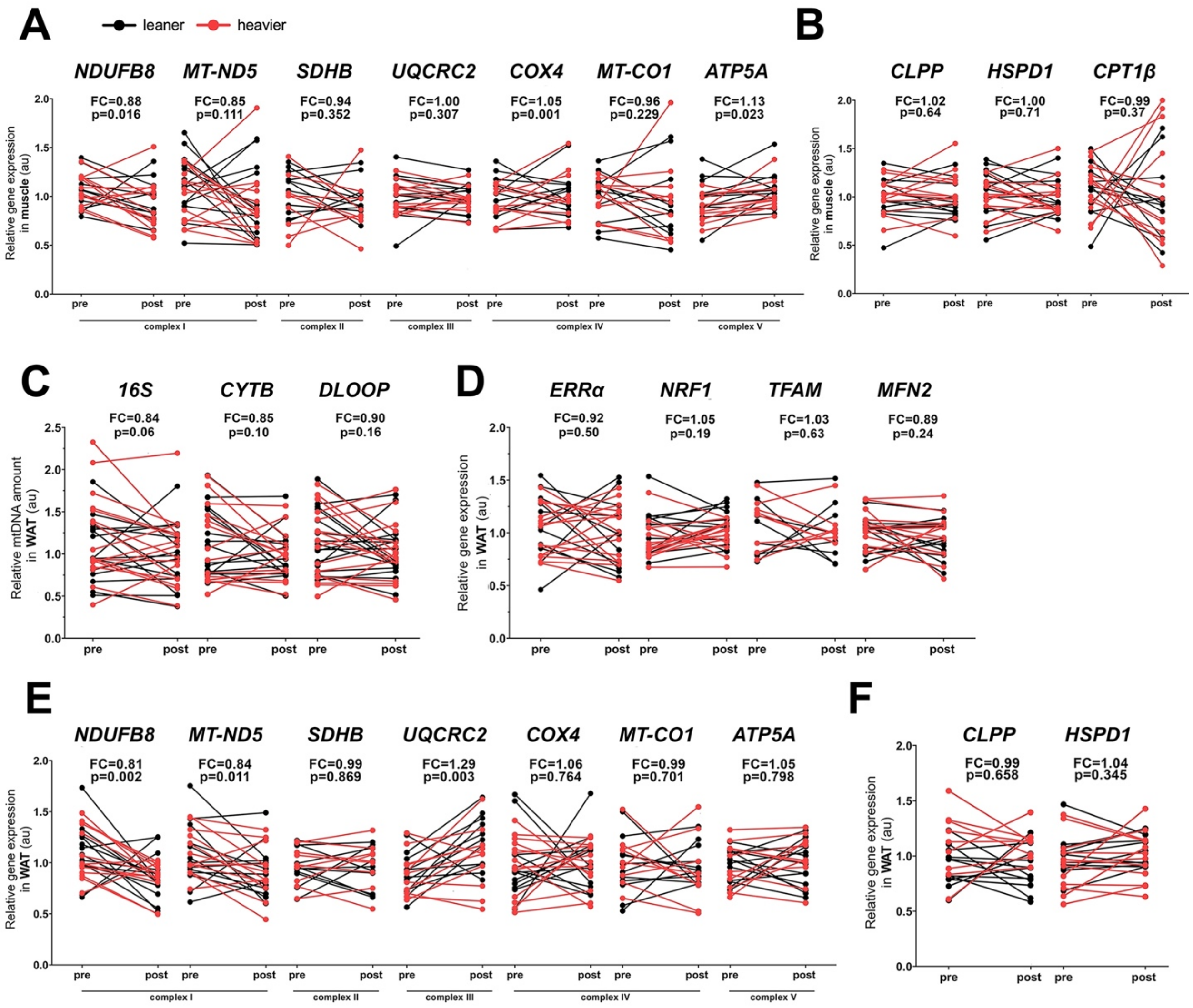

Fig. S3. Expression of mitochondrial metabolism-related genes in muscle and WAT and WAT mitochondrial DNA amount before and after NR treatment in the twins from the BMI-discordant pairs. Related to Fig. 2. Number of analyzed study participants is presented as n=number of full twin pairs/number of individuals.

(A) Gene expression of OXPHOS complex (I-V) subunits in muscle pre versus post NR (n= 10-13 pairs/21-26 individuals).

(B) Expression of mitochondrial quality control and fatty acid oxidation genes in muscle pre versus post NR (n= 11-13 pairs/23-26 individuals).

(C) Mitochondrial DNA amount in WAT pre versus post NR (n=14-15 pairs/28-31 individuals).

(D) Gene expression levels of the regulators of mitochondrial biogenesis in WAT pre versus post NR (n=10-13 pairs/21-27 individuals).

(E) Gene expression levels of OXPHOS complex (I-V) subunits in WAT pre vs post NR (n=9-13 pairs/19-26 individuals).

(F) Expression levels of mitochondrial quality control genes in WAT pre vs post NR (n= 11 pairs/22-23 individuals).

Pre and post value of each individual is connected with a line. The leaner cotwins are shown in black, the heavier cotwins in red. Fold change (FC) indicates the mean of the post NR values divided by the pre NR values. Paired Wilcoxon signed-rank test was used as the statistical test.

Arbitrary unit (au) indicates relative gene expression normalized to the expression of reference genes or mitochondrial amount expressed relative to nuclear genome amount. *NDUFB8*, NADH:ubiquinone oxidoreductase subunit B8; *MT-ND5*, mitochondrially encoded NADH dehydrogenase 5; *SDHB*, succinate dehydrogenase complex iron sulfur subunit B; *UQCRC2*, ubiquinol-cytochrome c reductase core protein 2; *MT-CO1*, mitochondrially encoded cytochrome c oxidase 1*; CLPP*, caseinolytic mitochondrial matrix peptidase proteolytic subunit; *HSPD1*, heat shock protein family D (Hsp60) member 1; *CPT1β,* carnitine palmitoyltransferase 1B; *16S*, 16 rRNA; *CYTB*, cytochrome b and *DLOOP*, D-loop region.

Table S1. The effect of NR on cardiovascular parameters in the twins from BMI-discordant pairs.
Data are shown as mean ± SD (normally distributed variables) or median (interquartile range, for skewed variables). P values were obtained using paired Wilcoxon signed-rank test to examine the effect of NR supplementation on clinical parameters in the twins from the BMI-discordant pairs (n=16 twin pairs/32 individuals). HDL, high-density lipoprotein; LDL, low-density lipoprotein; FFA, free fatty acids and BP, blood pressure.

**Variable (unit)**	**All Pre (*n*=32 individuals)**	**All Post (*n*=32 individuals)**	**P Value**	**Leaner Pre (*n*=16 individuals)**	**Leaner Post (*n*=16 individuals)**	**P Value**	**Heavier Pre (*n*=16 individuals)**	**Heavier Post (*n*=16 individuals)**	**P Value**
Cholesterol (total, mmol/l)	4.7 ± 0.8	4.7 ± 0.8	0.346	4.7 ± 0.7	4.7 ± 0.7	0.568	4.7 ± 0.9	4.8 ± 1.0	0.470
HDL (mmol/l)	1.3 (1.2 - 1.6)	1.2 (1.0 - 1.6)	0.049	1.4 (1.2 - 1.5)	1.3 (1.1 - 1.7)	0.093	1.3 (1.1 - 1.7)	1.2 (1.0 - 1.6)	0.289
LDL (mmol/l)	3.0 ± 0.8	3.0 ± 0.5	0.723	3.0 ± 0.7	3.0 ± 0.5	0.752	3.1 ± 0.8	3.0 ± 0.5	0.887
Triglycerides (mmol/l)	1.0 (0.7 - 1.2)	0.9 (0.8 - 1.3)	0.046	0.81 (0.6 - 1.0)	0.9 (0.7 - 1.1)	0.313	1.0 (0.7 - 1.3)	1.1 (0.8 - 1.4)	0.088
FFA (μmol/l)	444 (376 – 551)	431 (368 – 546)	0.229	415 (362 - 500)	392 (322 – 432)	0.561	510 (428 - 700)	483 (420 – 562)	0.252
Systolic BP (mmHg)	133 ± 22	128 ± 19	0.531	131 ± 24	124 ± 19	0.683	135 ± 22	131 ± 19	0.753
Diastolic BP (mmHg)	83 ± 12	83 ± 11	0.875	83 ± 12	81 ± 11	0.889	84 ± 11	84 ± 12	1.000
Pulse (per min)	67 ± 11	67 ± 9	0.629	65 ± 9	66 ± 9	0.753	69 ± 12	69 ± 9	0.806
Hs-CRP (mg/l)	1.9 (0.7 - 3.9)	1.6 (0.6 - 4.0)	0.655	1.7 (0.6 - 3.0)	1.1 (0.5 - 2.9)	0.561	2.3 (0.7 - 5.8)	2.5 (0.9 - 4.7)	0.978
Total homocysteine (μmol/l)	11.0 (9.30 -12.0)	12.0 (10.0 - 14.0)	0.005	10.0 (9.55 -12.0)	12.0 (10.5 - 14.0)	0.003	11.0 (9.28 -12.8)	11.5 (10.3 - 14.0)	0.551

Table S8. The effect of 5-month placebo and NR treatment in the twins from the BMI-concordant pairs.
Data are shown as mean ± SD (normally distributed variables) or median (interquartile range, for skewed variables). P values were obtained using paired Wilcoxon signed-rank test to examine the effect of placebo and NR treatment on anthropometric and clinical parameters in the twins from the BMI-concordant pairs (n= 4 pairs/8 individuals). HbA1c, hemoglobin A1c; HOMA, homeostasis model assessment; AUC, area under curve; OGTT, oral glucose tolerance test; HDL, high-density lipoprotein; LDL, low-density lipoprotein; FFA, free fatty acids; BP, blood pressure; AST, aspartate transaminase; MCV, mean corpuscular volume; MCH, mean corpuscular hemoglobin; MCHC, mean corpuscular hemoglobin concentration; fP, fasting plasma and Fe, iron.

**Variable (unit)**	**Placebo Pre (*n*=4 individuals)**	**Placebo Post (*n*=4 individuals)**	**P Value**	**Treated Pre (*n*=4 individuals)**	**Treated Post (*n*=4 individuals)**	**P Value**
BMI (kg/m^2)	31.5 ± 6.8	32.6 ± 7.3	0.125	32.0 ± 6.9	32.1 ± 6.7	0.875
Weight (kg)	87.2 ± 12.2	90.2 ± 13.2	0.125	89.1 ± 16.6	89.8 ± 15.2	0.581
Waist-hip ratio	0.9 ± 0.0	1.0 ± 0.0	0.250	0.9 ± 0.0	1.0 ± 0.1	0.250
Body fat (%)	34.1 ± 13.8	38.0 ± 12.3	0.250	33.2 ± 14.8	35.8 ± 13.3	0.750
Visceral adipose tissue (cm^3)	2017 ± 497	2275 ± 521	0.125	2217 ± 921	2171 ± 861	0.625
Subcutaneous adipose tissue (cm^3)	7297 ± 4799	7568 ± 4950	0.125	6996 ± 4831	6833 ± 4751	0.375
Adipocyte cell number (per billion)	233 (197 - 308)	289 (235 - 325)	1.000	168 (147 - 204)	315 (246 - 396)	0.125
Adipocyte cell diameter (μM)	89 ± 11	99 ± 8.6	0.375	96 ± 12	90 ± 5.6	0.125
Adipocyte cell volume (pL)	500 ± 154	734 ± 161	0.250	617 ± 219	573 ± 123	0.625
Adipocyte cell weight (ng)	407 (364 - 500)	702 (589 - 785)	0.250	584 (427 - 722)	559 (495 - 587)	0.625
Liver fat (%)	5.2 (0.87 - 9.9)	6.5 (1.5 - 12.0)	0.181	3.4 (2.0 - 5.4)	3.1 (1.9 - 4.8)	0.125
Lean tissue mass (kg)	56.2 ± 5.5	53.0 ± 8.8	0.750	58.8 ± 4.9	54.3 ± 9.7	1.000
Total bone mass (kg)	3.2 ± 0.6	3.0 ± 0.7	0.500	3.3 ± 0.5	3.0 ± 0.7	0.250
Basal metabolic rate (kcal/day)	1782 ± 266	1813 ± 260	0.250	1814 ± 298	1842 ± 262	0.125
Caloric intake (kcal/day)	1872 ± 419	1423 ± 730	0.250	2134 ± 883	1831 ± 1120	0.250
Protein intake (g)	81.5 (63.2 - 100.2)	89.5 (61.6 - 91.8)	1.000	84.0 (65.7 - 110.1)	69.2 (47.3 - 89.9)	0.125
Fat intake (g)	69.8 ± 17.5	52.7 ± 29.8	0.250	87.1 ± 39.5	70.2 ± 53.2	0.250
Carbohydrate intake (g)	203.5 ± 44.4	134.9 ± 66.8	0.500	221.2 ± 93.2	187.0 ± 131.5	0.625
Niacin equivalent (mg/day)	32.5 (30.6 - 36.5)	40.9 (29.1 - 44.1)	1.000	35.8 (31.9 - 41.6)	29.9 (21.7 - 37.7)	0.125
Physical activity (Total Baecke)	8.1 ± 0.7	8.3 ± 1.5	0.500	8.0 ± 1.7	8.0 ± 1.5	1.000
Alcohol intake (doses/week)	1.0 (0.5 - 1.4)	0.0 (0.0 – 7.0)	1.000	2.0 (0.0 - 4.6)	0.0 (0.0 - 2.5)	1.000
Current smoker (number)	2	1	-	1	1	-
HbA1c (mmol/mol)	34.5 (33.0 – 36.3)	35.5 (34.5 – 36.3)	1.000	36.5 (35.0 – 37.8)	37.0 (35.5. – 38.5)	0.371
Fasting glucose (mmol/l)	5.4 (5.0 - 5.8)	5.4 (5.2 - 5.6)	0.850	5.8 (5.6 - 5.4)	5.4 (5.2 - 5.5)	0.625
Fasting insulin (mIU/l)	7.7 (3.9 - 11.5)	6.1 (.3 - 11.9)	0.875	9.7 (4.1 - 14.9)	10.4 (4.4 - 16.2)	0.125
Fasting C-peptide (nmol/l)	0.7 (0.4 – 1.0)	0.6 (0.4 - 1.0)	0.875	0.8 (0.4 - 1.2)	0.8 (0.4 - 1.1)	0.250
HOMA index	1.8 (1.0 - 2.7)	1.5 (0.8 - 2.8)	0.875	2.5 (1.0 - 3.8)	2.4 (1.0 - 4.0)	1.000
Matsuda index	8.8 ± 6.9	6.9 ± 5.0	0.250	7.5 ± 6.5	5.3 ± 4.5	0.125
Glucose AUC during OGTT	14.8 ± 4.1	15.4 ± 3.2	0.875	16.8 ± 3.3	24.4 ± 13.1	0.625
Insulin AUC during OGTT	90 (30 - 161)	107 (49 - 190)	0.125	115 (39 - 188)	108 (45 - 200)	0.625
Adiponectin (μg/ml)	3.4 (2.8 - 6.8)	3.2 (2.4 - 6.9)	0.750	5.2 (3.6 - 6.9)	5.0 (3.7 - 6.2)	1.000
Cholesterol (total, mmol/l)	4.7 ± 0.5	4.9 ± 0.5	0.181	4.8 ± 0.8	4.7 ± 0.5	0.371
HDL (mmol/l)	1.3 (1.2 - 1.4)	1.4 (1.3 - 1.4)	0.875	1.4 (1.2 - 1.6)	1.3 (1.1 - 1.4)	0.098
LDL (mmol/l)	3.1 ± 0.41	3.4 ± 0.57	0.174	3.3 ± 0.45	3.4 ± 0.47	0.174
Triglycerides (mmol/l)	1.0 (0.9 - 1.0)	1.0 (0.8 - 1.3)	0.875	0.9 (0.7 - 1.2)	0.9 (0.8 - 1.1)	1.000
FFA (μmol/l)	427.7 (325.4 - 531.4)	415.3 (347.7 - 485.2)	0.625	394.1 (332.1 - 481.9)	456.3 (346.6 - 551.1)	0.625

Systolic BP (mmHg)	114 ± 12	122 ± 11	0.250	126 ± 8	129 ± 16	0.625
Diastolic BP (mmHg)	74 ± 9	80 ± 12	0.125	80 ± 5	82 ± 13	0.713
Pulse (per min)	63 ± 8	63 ± 13	1.000	61 ± 5	61 ± 13	0.875
Hs-CRP (mg/l)	1.6 (1.3 - 11)	2.0 (0.9 - 5.5)	0.250	1.0 (0.6 - 3.3)	0.4 (0.4 - 5.1)	1.000
Total homocysteine (μmol/l)	13.0 (12.2 - 13.8)	14.0 (12.1 - 15.8)	0.414	12.5 (11.5 - 15.0)	13.5 (12.1 - 17.0)	0.198
ALT (U/l)	24 (18 - 39)	24 (21 - 33)	1.000	23 (22 - 28)	27 (21 - 34)	1.000
AST (U/l)	27 (25 - 30)	23 (22 - 26)	0.250	27 (24 - 30)	31 (26 - 34)	0.581
Creatinine (μmol/l)	74 ± 13	75 ± 8	0.875	79 ± 12	76 ± 5	0.625
Hemoglobin (g/l)	137 ± 10	132 ± 6	0.197	136 ± 13	130 ± 12	0.197
Haematocrit (%)	40.8 ± 2.9	39.5 ± 2.1	0.173	40.5 ± 3.9	38.5 ± 3.0	0.174
Erythrocytes ($x10^{12}$/l)	4.5 ± 0.4	4.5 ± 0.3	0.875	4.6 ± 0.5	4.5 ± 0.4	0.250
MCV (fl)	90.3 ± 2.1	88.3 ± 3.9	0.250	87.8 ± 4.2	86.3 ± 5.6	0.346
MCH (pg)	30.5 ± 1.0	29.8 ± 0.96	0.149	29.8 ± 1.7	29.0 ± 2.2	0.371
MCHC (g/l)	338.0 (335.5 - 339.8)	335.0 (332.5 - 338.0)	1.000	338.5 (334.5 - 342.5)	335.5 (331.3 - 339.3)	0.625
Leukocytes ($x10^{9}$/l)	7.3 ± 3.0	7.0 ± 2.4	0.875	6.2 ± 1.7	6.0 ± 0.9	1.000
Thrombocytes ($x10^{9}$/l)	234 ± 29	260 ± 48	0.197	260 ± 53	238 ± 31	0.375
fP-Fe (μmol/l)	20.0 ± 6.4	20.4 ± 3.2	1.000	20.0 ± 9.1	16.6 ± 5.1	0.875

Table S9. The effect of NR on safety parameters in the twins from BMI-discordant pairs.
Data are shown as mean ± SD (normally distributed variables) or median (interquartile range, for skewed variables). P values were obtained using paired Wilcoxon signed-rank test to examine the effect of NR supplementation on safety measures in the twins from the BMI-discordant pairs (n=16 twin pairs/32 individuals). AST, aspartate transaminase; MCV, mean corpuscular volume; MCH, mean corpuscular hemoglobin; MCHC, mean corpuscular hemoglobin concentration; fP, fasting plasma and Fe, iron.

**Variable (unit)**	**All**	**All**	**P Value**	**Leaner**	**Leaner**	**P Value**	**Heavier**	**Heavier**	**P Value**
	**Pre (*n*=32 individuals)**	**Post (*n*=32 individuals)**		**Pre (*n*=16 individuals)**	**Post (*n*=16 individuals)**		**Pre (*n*=16 individuals)**	**Post (*n*=16 individuals)**	
ALT (U/l)	22 (18 - 43)	26 (17 - 43)	0.674	20 (17 - 34)	26 (19 - 36)	0.041	33 (20 - 49)	27 (17 - 44)	0.155
AST (U/l)	24 (22 - 31)	25 (21 - 30)	0.626	24 (20 - 29)	24 (21 - 35)	0.081	24 (23 - 31)	25 (22 - 28)	0.293
Creatinine (μmol/l)	74 ± 13	74 ± 12	0.650	73 ± 14	75 ± 12	0.551	74 ± 13	74 ± 12	0.924
Hemoglobin (g/l)	142 ± 9	140 ± 9	0.043	142 ± 8	140 ± 8	0.312	143 ± 9	141 ± 10	0.073
Haematocrit (%)	42.0 ± 2.2	42.1 ± 2.2	0.641	41.9 ± 2.1	42.4 ± 1.9	0.212	42.1 ± 2.4	41.9 ± 2.6	0.668
Erythrocytes (x10^{12}/l)	4.8 ± 0.3	4.8 ± 0.3	0.991	4.8 ± 0.37	4.8 ± 0.32	0.244	4.8 ± 0.31	4.8 ± 0.31	0.268
MCV (fl)	87.9 ± 3.5	88.0 ± 3.3	0.829	88.4 ± 4.2	88.3 ± 3.7	1.000	87.4 ± 2.6	87.7 ± 2.9	0.681
MCH (pg)	29.8 ± 1.3	29.4 ± 1.4	0.013	29.9 ± 1.5	29.3 ± 1.7	0.009	29.6 ± 1.2	29.6 ± 1.1	0.766
MCHC (g/l)	338 (334 - 342)	333 (330 - 340)	0.005	338 (334 - 342)	332 (327 - 338)	0.010	337 (335 - 342)	336 (331 - 341)	0.300
Leukocytes (x10^9/l)	6.4 ± 1.5	6.3 ± 1.8	0.134	6.5 ± 1.8	6.3 ± 2.0	0.277	6.3 ± 1.2	6.3 ± 1.7	0.244
Thrombocytes (x10^9/l)	243 ± 55	248 ± 62	0.199	244 ± 60	252 ± 72	0.301	241 ± 52	244 ± 52	0.569
fP-Fe (μmol/l)	19.0 ± 6.9	16.7 ± 5.9	0.078	19.8 ± 7.6	16.6 ± 5.9	0.084	18.1 ± 6.3	16.7 ± 6.1	0.349

Table S10. The primer sequences.

Gene	Gene/target name	Forward (5' - 3')	Reverse (5' - 3')
16S	16S rRNA mitochondrial genomic area	GGGGCGACCTCGGAGCAGAA	ATAGCGGCTGCACCATCGGGA
CYTB	Cytochrome B mitochondrial genomic area	GCCTGCCTGATCCTCCAAAT	AAGGTAGCGGATGATTCAGCC
DLOOP	12S rRNA mitochondrial genomic area	CATCTGGTTCCTACTTCAGGG-3'	CCGTGAGTGGTTAATAGGGTG
APP	Amyloid beta precursor protein nuclear genomic area	TGTGTGCTCTCCCAGGTCTA	CAGTTCTGGATGGTCACTGG
B2M	Beta-2-microglobulin nuclear genomic area	TGCTGTCTCCATGTTTGATGTATCT	TCTCTGCTCCCCACCTCTAAGT
HPP	Hemoglobin subunit beta nuclear genomic area	CAGGTACGGCTGTCATCAGTTAG	CATGGTGTCTGTTTGAGGTTGCT
ACTB	Actin	GATGAGATTGGCATGGCTTT'	CACCTTCACCGTTCCAGTTT
ATP5A	ATP synthase α	ATGACGACTTATCCAAACAGGC	CGGGAGTGTAGGTAGAACACAT
B2M	Beta-2-microglobulin	GAGGCTATCCAGCGTACTCCA	CGGCAGGCATACTCATCTTTT
CLPP	Caseinolytic mitochondrial matrix peptidase proteolytic subunit	CCATCTACGACACGATGCAG	CATGATCTCCTCTGCCTGA
COX4	Cytochrome c oxidase subunit 4	GGTTTCACCGCGCTCGTTAT	TGTCCAGCATCCTCTTGGTCTG
CPT1β	Carnitine palmitoyltransferase 1B	CATGTATCGCCGTAAACTGGAC	TGGTAGGAGCACATAGGCACT
ERRα	Estrogen-related receptor α	TATGGTGTGGCATCCTGTG	GTCTCCGCTTGGTGATCTC
HSPD1	Heat shock protein family D (Hsp60) member	CCAAGGAAGGCTTCGAGAAGAT	CGTAGCAACCTGTGCAATTTCT
MFN2	Mitofusin 2	GGCCCAACTCTAAGTGCCC	AAGTGCTTTTCCGTCTGCATC
MT-ND5	Mitochondrially encoded NADH: ubiquinone oxidoreductase core subunit 5	AGGCGCTATCACCACTCTGTTCG	AACCTGTGAGGAAAGGTATTCCT
MT-COX1	Mitochondrially encoded cytochrome c oxidase I	GGATGCATACACCACATGAA	AGCGAAGGCTTCTCAAATCA
MYF5	Myogenic factor 5	TCACCTCCTCAGAGCAACCT	ATTAGGCCCTCCTGGAAGAA
MYMK	Myomaker, myogenic fusion factor	GACAAGAGCGTCTACACCCA	AAGTGTAGTCCCAGTCCTCAAAG
MYOD	Myoblast determination protein 1	CCGCTTTCCTTAACCACAAA	CAAAGTGCTGGCAGTCTGAA
MYOG	Myogenin	GCCAGACTATCCCCTTCCTC	GAGGCCGCGTTATGATAAAA

NAMPT	Nicotinamide phosphoribosyltransferase	CTTCGGTTCTGGTGGAGGTT	TGTTGGGATCAGCAACTGGG
NAPRT	Nicotinic acid phosphoribosyltransferase	GACAGTGGTGACCTGCTACA	ACTGGTGCCAATGCCAATGA
NDUFB8	NADH:biquinone oxidoreductase subunit B8	ACAGGAACCGTGTGGATACAT	CCCCACCCAGCACATGAAT
NMNAT3	Nicotinamide-nucleotide adenylyltransferase 3	GTTGCTGAGCCCTGCAAATAG	GTGGCCACCCTGCTTATTAATTG
NRF1	Nuclear respiratory factor 1	AGGAACACGGAGTGACCCAA	TATGCTCGGTGTAAGTAGCCA
NRK1	Nicotinamide riboside kinase 1	TTTGGGAGGACTCGAACTGG	CACTGTTTGTCACACCACTGAT
PAX3	Paired box 3	GGCTTTCAACCATCTCATTCCCG	GTTGAGGTCTGTGAACGGTGCT
PAX7	Paired box 7	GGAGGATGAAGCGGACAAGAAG	AGGTCAGGTTCCGACTCCACAT
PGC1α	Peroxisome proliferator-activated receptor gamma coactivator 1α	AGCCTCTTTGCCCAGATCTT	GGCAATCCGTCTTCATCCAC
PPAR γ	Peroxisome proliferator activated receptor gamma	TACTGTCGGTTTCAGAAATGAC	GTCAGCGGACTCTGGATTCAG
SDHB	Succinate dehydrogenase complex iron sulfur subunit B	GCAGTCCATAGAAGAGCGTGAG-	TGTCTCCGTTCCACCAGTAGCT
SIRT1	Sirtuin 1	AGGCCACGGATAGGTCCATA	GTGGAGGTATTGTTTCCGGC
SIRT3	Sirtuin 3	ACCCAGTGGCATTCCAGAC	GCTTGGGGTTGTGAAAGAAGAA
TFAM	Transcription factor A	CCGAGGTGGTTTTCATCTGT	ACGCTGGGCAATTCTTCTAA
UQCRC2	Ubiquinol-cytochrome c reductase core protein 2	CCGTGGAATTGAAGCAGTTGGTG	CTGTGGTGACATTGAGCAGGAAC
YWHAZ	Tyrosine 3-monooxygenase/tryptophan 5-monooxygenase activation protein zeta	ACTTTTGGTACATTGTGGCTTCAA	CCGCCAGGACAAACCAGTAT
	Faecalibacterium prausnitzii genomic area	GAGCCTCAGCGTTGGTT	CCATGAACTGACTGTT
16S	Gut microbiota 16S rRNA genomic area	GTGYCAGCMGCCGGGGTAA	GGACTACNVGGGTWTCTAAT

Table S2 (Excel file). Differential methylation analysis comparing the response to NR supplementation within BMI-discordant twin pairs in muscle (n=12 pairs/24 individuals) and in WAT (n=14 pairs/28 individuals) (WAT starting row 624). CpGs that were included in the analysis annotate to the selected list of mitochondrial and NAD+ metabolism-related genes. A CpG was considered differentially methylated when FDR<0.05. The model was adjusted for age, sex, smoking, heavy/lean status and family relatedness. Related to Fig. 4.

Table S3 (Excel file). Associations between CpG methylation and gene expression upon NR supplementation in muscle (n ranges from 7 to 11 full pairs/15 to 23 individuals) and in WAT (n ranges from 10 to 12 full pairs/21 to 25 individuals) in the twins from the BMI-discordant pairs (WAT starting row 566). ß values are standardized regression coefficients and represent the change in CpG methylation according to the change in gene expression upon NR. The regression model was adjusted for sex, age, smoking status and family relationship. Related to Fig. 4.

Table S4 (Excel file). Metabolite abundances that were different after NR supplementation in the twins from the BMI-discordant pairs (n=14 pairs/28 individuals), nominal p<0.05. The regression model was adjusted for age, sex, smoking, heavy/lean status and family relatedness. Related to Fig. 5.

Table S5 (Excel file). Changes in the metabolite abundances that were different after NR supplementation between the leaner and the heavier cotwins from the BMI-discordant pairs (n=14 pairs/28 individuals), nominal p value <0.05. The regression model was adjusted for age, sex, smoking, heavy/lean status and family relatedness. Related to Fig. 5.

Table S6 (Excel file). Changes in the microbiota abundances after NR supplementation between the leaner and the heavier cotwins from the BMI-discordant pairs (n=11 pairs/22 individuals). The regression model was adjusted for age, sex, smoking, heavy/lean status and family relatedness. Related to Fig. 6.

Table S7 (Excel file). Associations of microbiota composition with NAD metabolites, blood clinical variables and plasma metabolites in the twins from the BMI-discordant pairs (n=11 pairs/22 individuals). ß values are standardized regression coefficients and represent i) the change in blood NAD metabolites according to the baseline microbiota composition and ii) the change in blood NAD metabolites, the change plasma clinical variables and the change in plasma metabolites according to the change in microbiota after NR treatment. The regression model was adjusted for sex, age, smoking status and family relationship. Related to Fig. 6.
